# Deep Sequencing Reveals Dual Evolution of SARS‐CoV‐2: Insights Into Defective Genomes From Wuhan‐Hu‐1 Variants to Omicron Subvariants

**DOI:** 10.1002/jmv.70476

**Published:** 2025-06-30

**Authors:** Carolina Campos, Marta Ibañez‐Lligoña, Sergi Colomer‑Castell, Josep Gregori, Damir Garcia‑Cehic, Cristina Andrés, Maria Piñana, Alejandra González‑Sánchez, Ariadna Rando‑Segura, Juliana Esperalba, Narcis Saubí, Maria Francesca Cortese, David Tabernero, Francisco Rodriguez‐Frias, Roser Ferrer, Juan Ignacio Esteban, Renate W. Kakze‐van der Honing, Andries A. Kampfraath, Júlia Vergara‐Alert, Joaquim Segalés, Wim H. M. van der Poel, Tomàs Pumarola, Andrés Antón, Josep Quer

**Affiliations:** ^1^ Liver Diseases‑Viral Hepatitis, Liver Unit, Vall d'Hebron Institut de Recerca (VHIR) Vall d'Hebron Hospital Universitari, Vall d'Hebron Barcelona Hospital Campus Barcelona Spain; ^2^ Centro de Investigación Biomédica en Red de Enfermedades Hepáticas y Digestivas (CIBERehd), Instituto de Salud Carlos III Madrid Spain; ^3^ Biochemistry and Molecular Biology Department Universitat Autònoma de Barcelona (UAB) Bellaterra Spain; ^4^ Medicine Department Universitat Autònoma de Barcelona (UAB) Bellaterra Spain; ^5^ Department of Radiation Oncology University of Cincinnati College of Medicine Cincinnati Ohio USA; ^6^ Respiratory Virus Unit, Microbiology Department Vall d'Hebron Hospital Universitari, Vall d'Hebron Barcelona Hospital Campus Barcelona Spain; ^7^ CIBER of Infectious Diseases (CIBERINFEC) Instituto de Salud Carlos III Madrid Spain; ^8^ Liver Unit, Microbiology Department, Vall d'Hebron Institut de Recerca (VHIR) Vall d'Hebron Hospital Universitari, Vall d'Hebron Barcelona Hospital Campus Barcelona Spain; ^9^ Basic Science Department International University of Catalonia Barcelona Spain; ^10^ Biochemistry Department Vall d'Hebron Hospital Universitari, Vall d'Hebron Barcelona Hospital Campus Barcelona Spain; ^11^ Clinical Biochemistry, Drug Delivery and Therapy (CB‐DDT) Research Group, Vall d'Hebron Institut de Recerca (VHIR), Vall d'Hebron Hospital Universitari, Vall d'Hebron Barcelona Hospital Campus Barcelona Spain; ^12^ Wageningen University and Research Lelystad the Netherlands; ^13^ Unitat Mixta d'Investigació IRTA‐UAB en Sanitat Animal, Centre de Recerca en Sanitat Animal (CReSA), Campus de la Universitat Autònoma de Barcelona (UAB), 08193 Bellaterra Barcelona Catalonia Spain; ^14^ IRTA Programa de Sanitat Animal, Centre de Recerca en Sanitat Animal (CReSA), Campus de la Universitat Autònoma de Barcelona (UAB), 08193 Bellaterra Barcelona Catalonia Spain; ^15^ Departament de Sanitat i Anatomia Animals, Facultat de Veterinària, UAB Bellaterra Cerdanyola del Vallès Spain; ^16^ Microbiology Department Universitat Autònoma de Barcelona (UAB) Bellaterra Spain

**Keywords:** deep‐sequencing, dual evolution, DVGs, Omicron, quasispecies

## Abstract

SARS‐CoV‐2 has evolved from early variants dominating the first (B.1.5, B.1.1) and second (B.1.177) pandemic waves, which exhibited a higher frequency of minority mutants with deletions leading to Defective Viral Genomes (DVGs) in the *spike* region near the S1/S2 cleavage site than the Alpha, Beta, and Delta variants. The emergence of Omicron has significantly altered the dominant variant profile, with Omicron subvariants now representing 100% of circulating viruses. To monitor the evolution and adaptation of Omicron in the human population, a deep‐sequencing study was performed in RNA samples of BA.1, BA.1.1, BA.2, BA.5, BQ.1.1, XBB.1.5 and BA.2.86 Omicron subvariants. The findings reveal two occurrences of similar evolutionary patterns within SARS‐CoV‐2 characterized by a shift from a significant to a very low production of DVGs. This event suggests that DVGs might play a role in the virus's spread and adaptation for persistence in infected humans.

## 1 Introduction

Severe acute respiratory syndrome coronavirus 2 (SARS‐CoV‐2) is the causal agent of millions of deaths since the first reported cases of the Coronavirus Disease 2019 (COVID‐19) in December 2019. Throughout the pandemic, thousands of variants and subvariants have emerged due to the rapid spread of the virus across the human population [1].

The SARS‐CoV‐2 Omicron variant has exhibited higher transmissibility [2] and greater resistance to vaccines due to a reduced effectiveness of vaccine‐induced immunity compared to other variants that dominated the first 2 years of the global COVID‐19 pandemic [3]. These factors contributed to the rapid spread of the virus.

The most remarkable mechanism of variability in RNA viruses is the lack of a proofreading enzyme and the low fidelity of the RNA‐dependent polymerase, leading to high mutation rates [4]. However, SARS‐CoV‐2 has a unique proofreading mechanism that avoids mismatches during replication [5], resulting in a lower mutation rate (1 × 10^‐6^–2 × 10^‐6^) compared to other RNA viruses, for instance, Hepatitis C virus or Human immunodeficiency virus (HIV) [6]. Therefore, the genomic variability of SARS‐CoV‐2 is attributed to many factors, including the extremely high number of infection events that have occurred in short periods, the intra‐host cell factors, reinfections of immune individuals, and long‐term infections in immunocompromised patients [6].

Genetic mutations are key drivers of viral evolution. Omicron emerged with 46 exclusive mutations within the whole genome [7]. Thirty signature mutations, two deletions, and one insertion specifically located in the Receptor Binding Domain (RBD) of Subunit 1 (S1) and Subunit 2 (S2) of the spike protein molecularly define the Omicron variant [8]. Some amino acid (aa) mutations are known to take place in the main epitopes of neutralizing antibodies (NAbs) or are directly involved in recognizing the hACE2 receptor, potentially contributing to the higher transmissibility of Omicron variant [2].

RNA viruses arrange mechanisms that interfere with the evolution of intercellular infection. A prominent example is the existence of defective viral genomes (DVGs) or defective interfering particles (DIPs) which are truncated, virus‐dependent particles unable to complete a full replication cycle [9]. These sub‐viral particles are produced as a side‐effect of point mutations, hyper‐mutations, frame‐shifts, deletions, and RNA recombination events [9]. DVGs trigger several cellular pathways by activating the antiviral immune response and potentially reducing disease severity in respiratory infections such as influenza [10].

DVGs contribute to the complexity and adaptability of viral populations and influence viral behavior by interfering with the replication of standard viruses [9]. For instance, DVGs in Respiratory Syncytial virus (RSV) can modulate immune responses helping viruses to evade the host's immune system by acting as decoys, facilitating persistence of the virus by creating a more favorable environment for viral replication [9, 11], and decreasing disease severity as shown in respiratory infections such as influenza [10]. DVGs can trigger strong innate immune responses that in some circumstances might benefit the spread or persistence of the virus [12, 13] and promote evolutionary advantages contributing to genetic diversity of a viral population giving for instance a better chance of overcoming host defenses [11].

In previous work, a higher frequency of defective deletions in the *spike* gene was reported, particularly close to the S1/S2 cleavage site, in variants that dominated the first (B.1.5, B.1.1) and the second (B.1.177) pandemic waves of 2020, compared to the Alpha, Beta, and Delta variants, which showed a smaller number of defective genomes [14]. Since the emergence of Omicron (B.1.1.529), the global profile of circulating SARS‐CoV‐2 variants has drastically changed, with Omicron subvariants now representing 100% of the circulating viruses [15]. Surprisingly, rather than Alpha, Beta, and Delta variants, the initial Omicron isolates showed a frequency of minority mutants with deletions in the S1/S2 region similar to the dominant variants from the first and second waves [14].

Hence, the objective of the present study was to monitor the evolution of DVGs in SARS‐CoV‐2 quasispecies by studying the complete spike gene from isolates of Omicron subvariants BA.1, BA.1.1, BA.2, BA.5, BQ.1.1, XBB.1.5, and BA.2.86. The discussion focuses on the changes in the presence and abundance of DVGs and their implications for the virus's spread and adaptation for persistence in infecting humans.

## 2 Materials and Methods

Nasopharyngeal swab specimens were collected from confirmed laboratory cases at Vall d'Hebron University Hospital from several primary care centres in Barcelona. Samples were randomly selected as in previous studies [14, 15] from mild and asymptomatic patients infected with Omicron subvariants in Barcelona city, from the 6th week of 2022 to the 46th week of 2023. These subvariants included BA.1, BA.1.1, BA.2, BA.5, BQ.1.1, XBB.1.5 and BA.2.86. Patient data was obtained retrospectively. The cycle threshold (Ct) values were not available due to SARS‐CoV‐2 detection performed by Cobas 5800 System SARS‐CoV‐2 Test (Roche Diagnostics, USA).

The institutional review board of the Clinical Research Ethics Committee (CEIm) from Vall d'Hebron University Hospital approved the study (PR(AG)259/2020). The need for informed consent was waived by CEIm Vall d'Hebron University Hospital, as the study used data routinely collected for surveillance activities. All methods were performed in accordance with relevant guidelines and regulations.

SARS‐CoV‐2 *spike* gene was retrotranscribed into cDNA by random priming using Random Hexamers (Integrated DNA Technologies, Iowa, USA) by the Super Script III reverse transcriptase (Thermo Fischer Scientific, Massachusetts, USA). The reaction conditions were as follows: incubation at 25°C for 5 min, followed by retrotranscription at 42°C for 50 min, and enzyme inactivation at 70°C for 10 min. The cDNA was then amplified using ARTIC v4.1 primers from the ARTIC Network. Primers from nCoV‐2019_72 to nCoV‐2019_84 generated overlapping amplicons ranging from 21533 to 25462 nucleotides (nt) (artic28‐ncov2019/nCoV‐2019.scheme.bed, ARTIC Network). *Spike* pair and impair primers testing for efficacy is specified in previous work [14]. Amplification was performed using two primer mixes in a final volume of 25 µL: a paired amplicons mix and a second impaired amplicons mix. The reaction was performed using the Q5 Hot‐start polymerase (New England BioLabs, Massachusetts, USA) with the following reaction program: hot‐start and dehybridization at 98°C for 30 s, polymerization at 98°C for 15 s per 35 cycles, and inactivation at 63°C for 5 min. Purification was executed using KAPA Pure Beads (KAPA Biosystems—Roche, Basel, Switzerland) in a 1:1× ratio of beads to sample, with elution in 30 µL of elution buffer (10 mM, Tris‐Cl). Final quantification was carried out by fluorometry using the dsDNA Broad Range kit from QUBIT3 technology (Thermo Fischer Scientific, Massachusetts, USA). Bands were checked using Tape Station Agilent technology following manufacturer's instructions (Agilent Technologies, Santa Clara, USA).

Seven libraries were prepared for SARS‐CoV‐2 spike gene amplicons sequencing using the KAPA Library HyperPrep kit (KAPA Biosystems—Roche, Basel, Switzerland) by using KAPA UDI adapters (KAPA Biosystems—Roche, Basel, Switzerland). Paired ends sequencing was performed using the MiSeq Reagent kit v3 for 600 cycles (Illumina, California, USA).

The aim of the bioinformatics analysis was to detect deletions in haplotypes, and they were performed as described in previous report [14].

## 3 Results

The study was performed on 92 nasopharyngeal exudate samples from COVID‐19 patients, with the following distribution: 13 infected by the BA.1 Omicron subvariant, 14 by BA.1.1, 14 by BA.2, 13 by BA.5, 15 by BQ.1.1, 15 by the XBB.1.5 recombinant subvariant, and 8 by the BA.2.86 subvariant (Supporting Information S1: Table 1). Sequences from the Omicron variants BA.1, BA.1.1, BA.2, BA.5, BQ.1.1, XBB.1.5, and BA.2.86 were uploaded to the Genebank Sequence Read Archive (SRA) database with BioProject accession number PRJNA1134434. Sequences from B.1.5, B.1.1, B.1.177, Alpha, Beta, and Delta variants were previously uploaded with accession number PRJNA788442, and sequences from Omicron B.1.1.529 BA.1 (12/2021) with accession number PRJNA1134434. A total of 106 358 172 reads were obtained (Supporting Information S1: Table 2]. Outliers over the mean from most amplicons in all subvariants are observed which means that excellent depths were reached in all runs, except for amplicon A75 from BA.2 and BQ.1.1 subvariants, where 7867–99 259 reads and 10 358–41 137 reads were obtained, respectively (Supporting Information S1: Table 2 and Figure 1A–G).

Figure 1Coverage by amplicon per Omicron subvariants (A) BA.1, (B) BA.1.1, (C) BA.2, (D) BA.5, (E) BQ.1.1, (F) XBB.1.5, and (G) BA.2.86.

**Figure:**
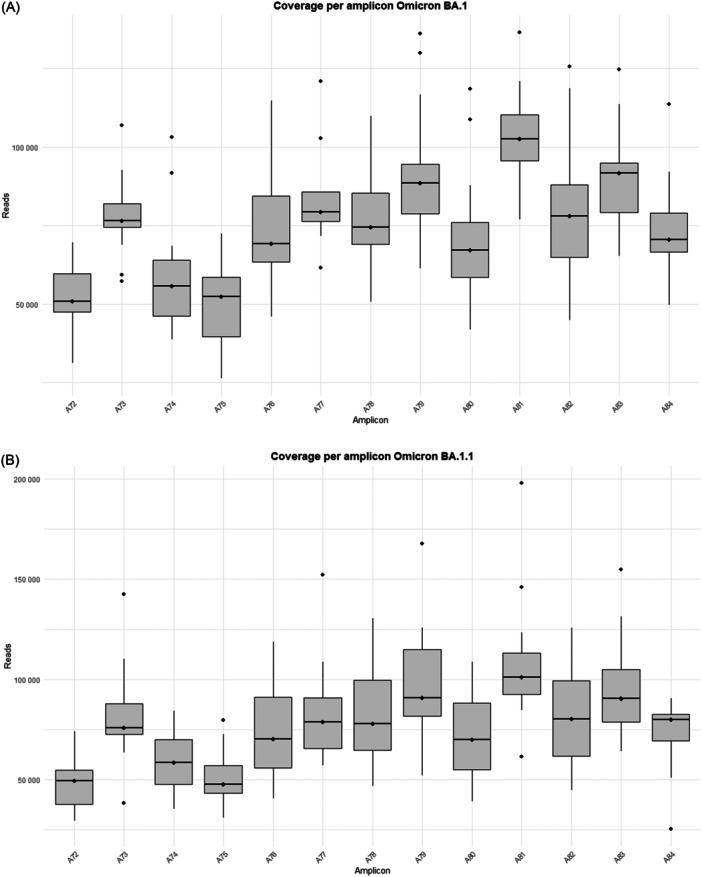

**Figure:**
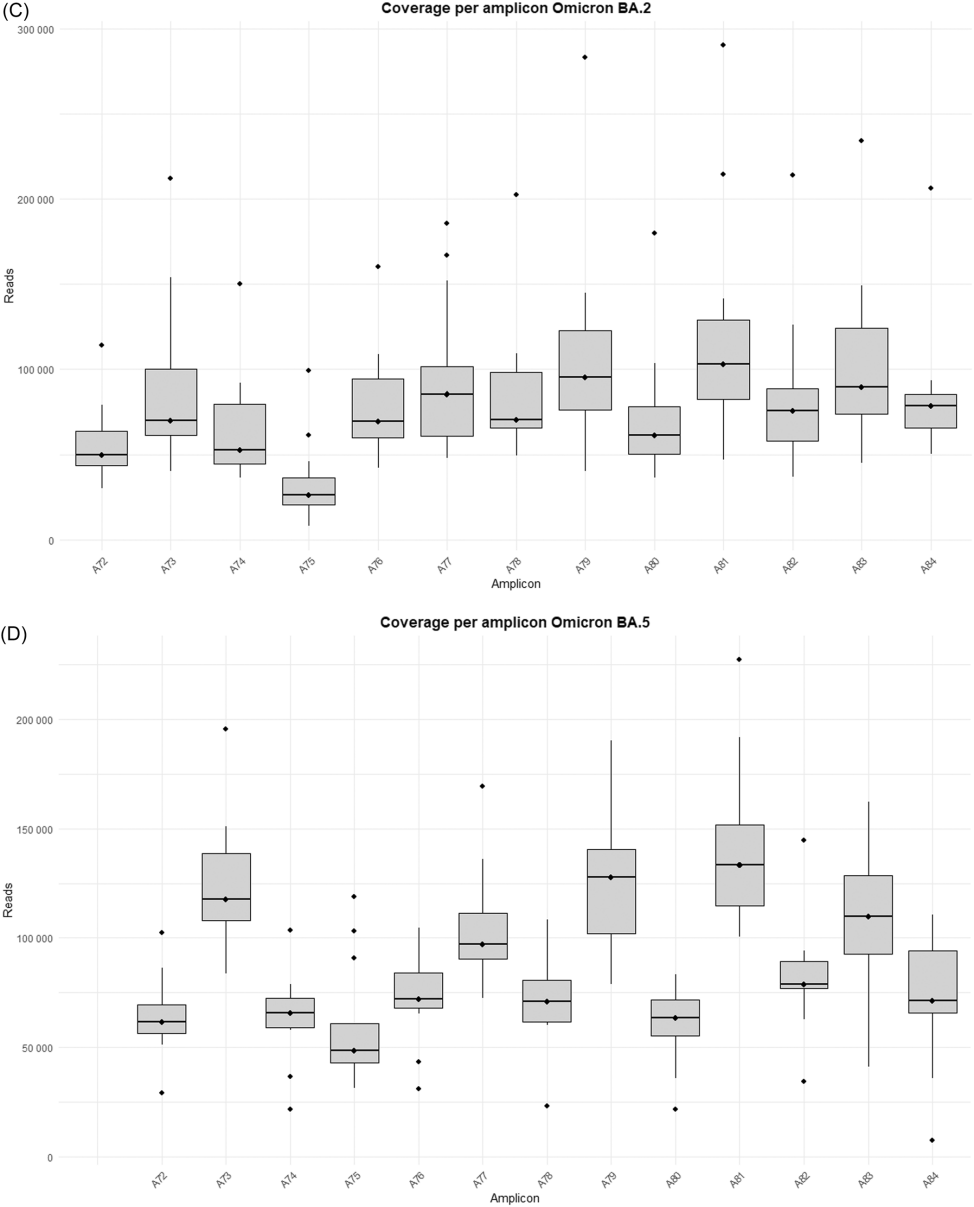

**Figure:**
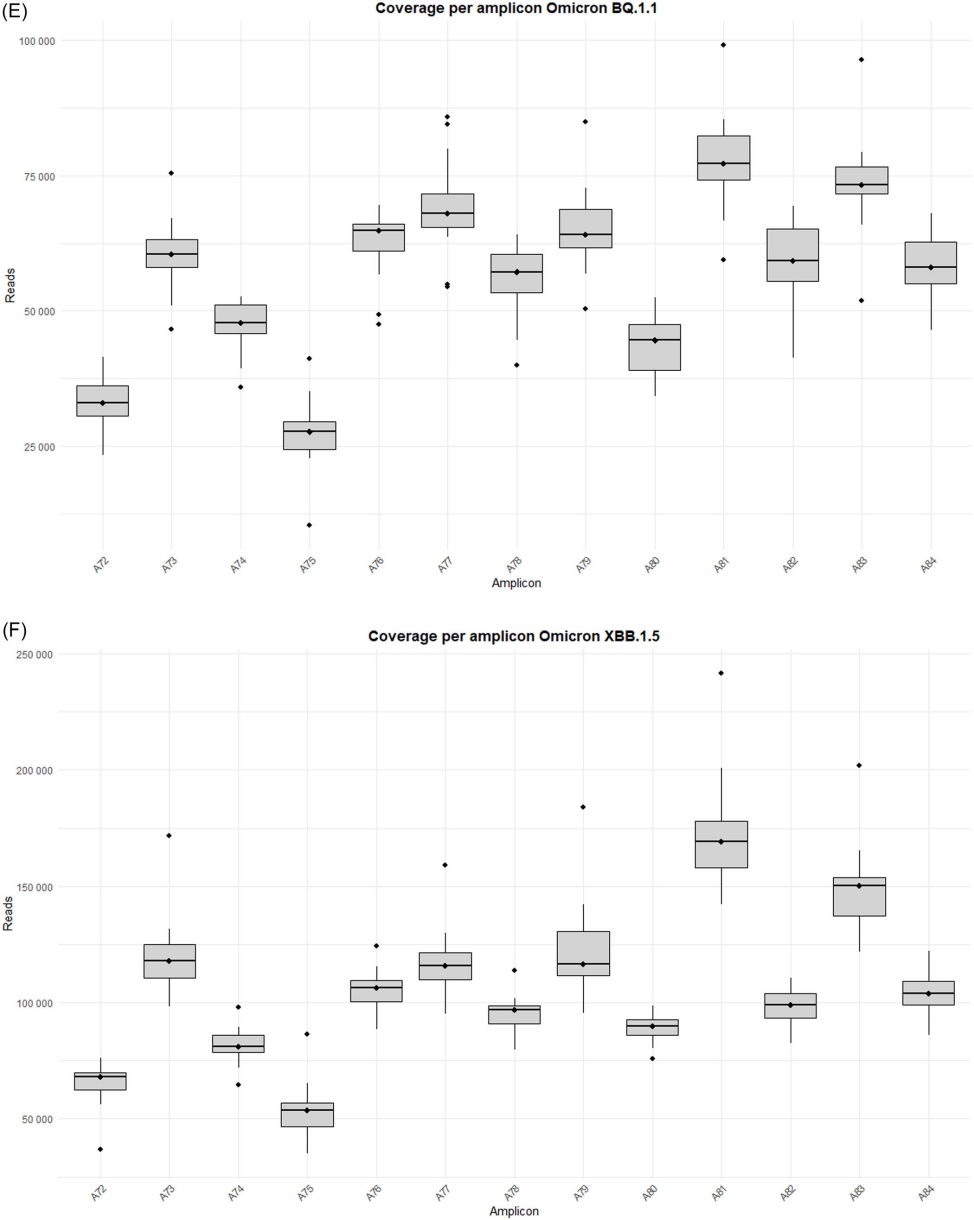

**Figure:**
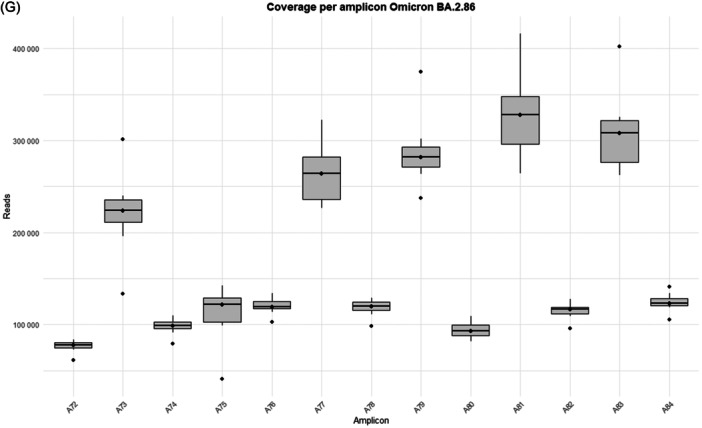

Thirty‐four out of 92 patients (37%) were found to have deletions throughout the spike gene, leading to a frameshift and a premature stop codon, consistent with previous findings [14]. Defective deletions per variant are described in Supporting Information S1: Table 3, where Omicron BA.1 12/2021 samples from the previous study are also included [14]. Among the patient population exhibiting these defective deletions, the initial Omicron BA.1 (02/2022) subvariant accounted for 38.5% (5 out of 13), followed by BA.1.1 at 50% (7 out of 14), BA.2 and BA.5 at 28.5% (4 out of 14) each, and BQ.1.1 at 6.7% (1 out of 15). The latest circulating variants BA.2.86 had defective genomes in 50% of patients (4 out of 8), and the recombinant XBB.1.5 Omicron subvariant accounted for 53.3% of patients (8 out of 15) with at least one defective genome detected, which was unexpected (Supporting Information S1: Table 3). However, all defective deletions found in XBB.1.5 subvariant mostly corresponded to a unique patient (P85) (Supporting Information S1: Table 3), except for the Δ145Y‐146H in amplicon A73, which was found in 8 out of 15 patients, representing 3.07% of population frequency (Table 1).

**Table 1:** Population frequency of defective haplotypes per variant. Δ = Deletion. Yellow cells denote the highest frequent defective deletions, green cells indicate deletions occurring in multiple variants, and orange cells mark the region of interest examined in this study.

Defective deletions	Omicron BA.1 (12/2021)	Omicron BA.1 (02/2022)	Omicron BA.1.1	Omicron BA.2	Omicron BA.5	Omicron BQ.1.1	Omicron XBB.1.5	Omicron BA.2.86
**Δ21R**		0.14%						
**Δ55F**			0.30%					
**Δ108T‐110/118 L**	0.41%							
**Δ110L**			0.2%	0.17%		0.19%	0.31%	0.17%
**Δ114T**								0.13%
**Δ115Q**					0.44%			
**Δ118L‐119I**							0.34%	
**Δ131C‐132E**		0.14%						
**Δ139P‐144Y**					0.48%			
**Δ145Y‐146H**							3.07%	
**Δ154E‐181G**			0.20%					
**Δ169E‐170Y**		0.17%						
**Δ193V‐194F**		0.22%						
**Δ194F‐203I**		0.31%						
**Δ196N‐203I**			0.20%					
**Δ199G‐200Y**			0.18%					
**Δ199G‐201F**								0.19%
**Δ210I‐212L**		0.82%	0.15%					
**Δ231I‐232G**				0.25%				
**Δ242L‐243A**				0.37%				
**Δ244L**								0.49%
**Δ244L‐245H**		0.28%						
**Δ246R‐249L**	7.03%							
**Δ286T‐287D**							0.44%	
**Δ289V‐290D**		0.16%						
**Δ294D**			0.26%					
**Δ346R**	0.23%							
**Δ361C**							0.36%	
**Δ368L**							0.30%	
**Δ393T**							0.27%	
**Δ396Y‐397A**	0.42%							
**Δ397A‐398D**	0.14%							
**Δ410I‐411A**					0.19%			
**Δ429F‐444K**								0.19%
**Δ433V‐434I**			0.14%					
**Δ456F‐468I**					0.19%			
**Δ467D**					0.15%			
**Δ474Q‐475A**	0.58%							
**Δ489Y‐491P**		0.16%						
**Δ508Y**		0.15%						
**Δ541F‐543K**						0.18%		
**Δ570A‐571D**		0.15%						
**Δ575A‐576V**			2.9%					
**Δ594G**	0.13%							
**Δ618T‐619E**								0.13%
**Δ621P‐622V**				0.17%				
**Δ626A‐629L**			0.12%					
**Δ640S/654E‐674Y**	0.69%	0.54%						
**Δ675Q**		0.36%						
**Δ744G**				0.13%				
**Δ805I**	0.19%							
**Δ817F‐821L**		0.35%	0.14%					
**Δ818I‐823F**				0.19%				
**Δ851C‐856N/873Y**	1.76%							
**Δ856N‐860V**			0.33%	0.18%	0.16%			
**Δ859T‐860V**		0.38%						
**Δ867D‐894L**	0.18%							
**Δ890A‐912T**	0.35%							
**Δ939S‐951V**								0.13%
**Δ1007Y‐1008V**	0.17%							
**Δ1006T‐1007Y**								0.18%
**Δ1016A‐1017E**							0.36%	
**Δ1028K**	0.66%							
**Δ1062F**	0.23%							
**Δ1074N‐1075F**				0.15%				
**Δ1075F‐1076T**	0.24%							
**Δ1087A‐1099G**	0.31%							
**Δ1133V**			0.25%					
**Δ1206Y‐1240C**								0.13%
SHARED								
REGION OF INTEREST								
HIGHEST FREQUENCY								

From Omicron BA.1 (12/2021) [14], there was a decrease in the recorded defective deletions but also in frequency. Remarkably, there was a punctual increase in the frequency of defective haplotypes in XBB.1.5, but as mentioned above, this was exclusive to a unique patient (Supporting Information S1: Table 3). In total, 53 new deletions resulting in defective haplotypes were identified (Table 1).

It is worth noting that defective deletions were identified in amplicon A78 from the Omicron BA.1 subvariant grouped in the same region. A notable occurrence of defective deletions is evident within the Δ654E‐674Y region (1970nt‐2029nt), aligns with the Δ640S‐674Y genomic location (1920nt‐2021nt), reported in previous research [14]. The Δ654E‐674Y defective deletion was detected in 0.54% of the Omicron BA.1 subvariant. Notably, these defective genomes reported in the Omicron BA.1 subvariant show concordance with previous results [14], identifying defective genomes in the Δ640S‐674Y region in the most widespread variants during the early stages of the pandemic (B.1.5, B.1.1 and B.1.177) as well as in Omicron BA.1 (12/2021). Surprisingly, none of these defective deletions were identified in any other Omicron subvariant in this study, including BA.1.1, BA.2, BA.5, BQ.1.1, XBB.1.5, and BA.2.86 (Table 1), although subvariant sequencing achieved similar high depth coverage (Figure 1A–G and Supporting Information S1: Table 2).

Some deletions are subvariant specific, while others are shared between subvariants, as in the case of the defective deletions Δ110L, Δ210I‐212L, Δ817F‐821L, and Δ856N‐860V (Table 1). Population frequencies were calculated as previously reported [14], and it is noteworthy that the three most common defective haplotypes are Δ145Y‐146H (3.07%) and Δ575A‐576V (2.9%), from Omicron XBB.1.5 and BA.1.1 respectively, and Δ210I‐212L (0.82%) and Δ654E‐674Y (0.54%), from Omicron BA.1.

**Figure 2:**
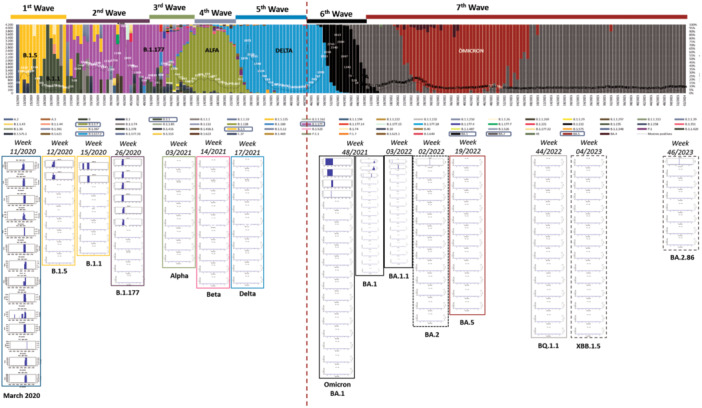
Weekly lineage distribution and positive samples detected (dashed line) from March 2020 to February 2024 in Barcelona, Spain. Bar plots below the lineage distribution display defective genomes starting from the initial cases identified in March 2020: B.1.5 (yellow), B.1.1 (dark green), B.1.177 (violet), Alpha (light green), Beta (pink), Delta (blue), Omicron BA.1 (black), Omicron BA.2 and Omicron BA.2‐like (grey) and Omicron BA.5 (red). These bar plots illustrate defective deletions in amplicon A78 across the 174 patients examined at the nucleotide level. Barr plots per amplicon and per variant from B.1.5 to Omicron BA.1 (December 2021) are collected in our previous report [16]. Barr plots from Omicron BA.1 (February 2022) to Omicron BA.2.86 are assembled in Supporting Material Figure 1A–G.

Some differences in the genomic location of the defective deletions were observed when comparing the variants. In the early‐onset Omicron BA.1 variant and its subsequent BA.1.1 and BA.2 variants, defective deletions were found in specific amplicons. However, in the later emerging BA.5 and BQ.1.1 subvariants, these defective deletions were only identified in specific amplicons: A73, A76, A77, and A80 (Supporting Information S1: Table 3; Supporting Information S1: Figure 1A–G). In contrast, the most recent Omicron subvariants included in this study (XBB.1.5 and BA.2.86) reveal an increase in the number of amplicons with defective genomes, but only three patients showed deletions in more than a single amplicon (P85, P87, and P88) (Supporting Information S1: Table 3; Supporting Information S1: Figure 1A–G). Furthermore, DVGs in the region of interest located in amplicon A78 were undetectable from Omicron BA.1.1 to Omicron BA.2.86 subvariants, in contrast to the Omicron BA.1 (02/2022) subvariant and previous SARS‐CoV‐2 variants [14]. It is also remarkable that four specific defective deletions (Δ817F‐821L, Δ818I‐823F, Δ856N‐860V and Δ859T‐860V) in amplicon A80 were detected in most Omicron subvariants belonging to the same genomic region (Table 1), excluding Omicron BQ.1.1, XBB.1.5 and BA.2.86 subvariants. Furthermore, Δ817F‐821L and Δ818I‐823F were previously detected as Δ817F‐822L and Δ818I‐822L [14] with only one amino acid disparity. A summary of the most important results of the present study is shown in Figure 2.

## 4 Discussion

In a previous work, an evolution of SARS‐CoV‐2 was reported, from variants dominating the first (B.1.5, B.1.1) and second (B.1.177) pandemic waves, which showed a higher frequency of minority mutants with deletions causing defective genomes in the *spike* region near the S1/S2 cleavage site, to variants (Alpha, Beta, and Delta) with a lower presence of deleted genomes [14]. It was hypothesized that, in the 2020 scenario of lacking immune protection against SARS‐CoV‐2 in humans, defective genomes might have favoured their spread by causing a lower severity of infection [14], thereby overcoming other variants. On the other hand, the reduction in defective genomes observed in the Alpha, Beta, and particularly the Delta variants could be associated with increased transmission and greater disease severity due to the higher production of infectious virions without affecting viral load compared to variants producing defective (noninfectious) genomes. In this sense, previous data are consistent with CDC reports concluding that Delta infection is associated with a higher risk of severe disease [14].

Unexpectedly, defective viral genomes were newly detected in the Omicron BA.1 (12/2021) [14] and BA.1 (02/2022) subvariants. The rapid spread of Omicron, which ultimately displaced Delta as the dominant variant, could be driven by its pronounced tropism for the upper respiratory tract, thereby enhancing viral transmissibility as previously reported [16]. After Omicron completely displaced earlier non‐Omicron variants, here the emerging Omicron subvariants (BA.1.1, BA.2, BA.5, XBB.1.5 and BA.2.86) show an identical evolution of DVGs as previously described in the 2020 dominant SARS‐CoV‐2 variant. Surprisingly, defective genomes remain undetected in the spike protein S1/S2 cleavage site in these emerging subvariants. This is consistent with the higher infectivity and transmissibility reported for these Omicron subvariants [17] and suggests that in an equal viral load context, more infective viruses are produced. It is worth mentioning that a more severe disease was described in unvaccinated patients infected by subsequent Omicron subvariants [18, 19]. This scenario is in concordance with the suggested parallel evolution of the Omicron subvariant rather than continuous evolution from previously dominant variants [3, 20]. Certainly, a double evolution pattern of SARS‐CoV‐2 is being confronted: from the Wuhan Hu‐1 virus to the Delta variant and from Omicron BA.1 (12/2021) and BA.1 (02/2022) to the BA.2.86 variant and the newly emerging SARS‐CoV‐2 viruses.

Previous reports demonstrate that cells accumulating defective particles create a competitive but balanced scenario between viral sub‐particles and full‐length viruses that compete for replication machinery [9]. A balanced ratio of DVGs vs. viral particles might enhance viral preservation by limiting host damage. Hence, the virus spares the host to ensure its survival [9, 10]. DVGs play an active role in pro‐survival and viral persistence of respiratory viruses, including RSV, Sendai virus (SeV), parainfluenza virus, and measles virus (MV) [11, 21, 22]. Certainly, in SeV infections, DVGs accumulation in cell cytoplasm determines two cellular subsets performing specific roles in viral infection [11, 22]. In addition, hotspots of copy‐back DVGs (cpDVGs) in SeV suggest that DVGs existence is a regulated process, rather than a random occurrence as previously thought [22].

This study describes a new evolutionary mechanism, that could explain the selection of more infective and transmissible viral variants. The virus initially produces defective particles, so that after viral spread and the population in contact with the initial variants increases, viruses with enhanced infectivity and transmissibility are selected over the defective ones. This mechanism is based on the loss of residues located before the S1/S2 cleavage site (682aa–685aa), after the RBD (involved in the cellular receptor recognition), and before the S2 region that is engaged in the viral entrance into the cellular citoplasma [23].

A key limitation of our study is the incomplete representation of all SARS‐CoV‐2 variants that have circulated globally throughout the pandemic. Although we included major variants of concern and interest, from early lineages (such as B.1.5, B.1.1 and B.1.177) to Alpha, Beta, and Delta variants; and Omicron B.1.1.529 and BA.1 to BA.1.1, BA.2, BA.5, BQ.1.1, XBB.1.5, BA.2.86, our ability to capture the full evolutionary landscape was limited by the availability of representative sequences, especially for transient or geographically restricted lineages. Additionally, the rapid and ongoing emergence of new variants posed challenges for exhaustive inclusion. As a result, some intermediate evolutionary steps may be underrepresented. Future studies leveraging more comprehensive, globally integrated genomic datasets will be critical to better resolve the full evolutionary dynamics of SARS‐CoV‐2.

The outlook of the circulating variants has widely changed over the 4 years of confronting the pandemic. Currently, the persistent Omicron BA.2.86 variant and especially its subvariant JN.1 dominate 100% of SARS‐CoV‐2 detections in primary care centres in Barcelona. This scenario suggests an extraordinary adaptation of the BA.2.86 subvariant, enabling it to surpass its competitors and overcome selective pressures such as vaccination and immunity acquired from previous SARS‐CoV‐2 infections [24, 25]. Reports describe a different antigenic profile, but also an enhanced virus‐cell membrane fusion process due to the P681R mutation in the spike protein [25].

In summary, besides these beneficial mutations, herein it is portrayed that viruses lacking DVGs have been selected throughout the evolution of Omicron since its emergence. These results demonstrate that SARS‐CoV‐2 has undergone a similar dual evolution, transitioning from variants producing significant DVGs to variants producing very few DVGs (Figure 2). The first round of evolution included early pandemic variants (B.1.5, B.1.1 and B,1,17) to Alpha, Beta, and Delta variants; and a second round from Omicron B.1.1.529 and BA.1 to BA.1.1, BA.2, BA.5, BQ.1.1, XBB.1.5, BA.2.86 (Figure 2). Thus, the overall landscape suggests that the loss of DVGs might have been beneficial for viral fitness by inducing a second phase of viral evolution. In addition, the reported background of DVGs generation and behaviour in other respiratory infections highlights the need for in vitro studies to elucidate the specific role of DVGs in SARS‐CoV‐2 infection and in vivo experiments to test DVGs effects over the immune system. In conclusion, defective genomes are involved in the evolution and adaptation of SARS‐CoV‐2 in the human population.

## Author Contributions

C.C., S.G.‐C., D.G.‐C., have contributed to the design of experiments and performing the technical work involving RNA extraction, amplification, and deep sequencing. J.G. and M.I.‐LL. developed software used in the study. C.C., C.A., M.P., J.G. designed the graphics. C.A., M.P., A.G‐S., A.R.‐S., J.E., N.S., R.F., M.F.C., D.T. collected samples and actively corrected the manuscript. J.S., J.V.‐A., R.W.K.‐V.D.H., A.A.K., W.H.M.V.D.P., F.R‐F., J.I.E., took an active role in discussing and correcting the draft. W.H.M.V.D.P., J.S., J.V.‐A., J.G., T.P., A.A., and J.Q. were involved in the data analysis and interpretation, and they thoroughly revised the draft. W.H.M.V.D.P., J.S., J.V.‐A., T.P., A.A., and J.Q. oversaw the data analysis, drafted the manuscript, and guided the discussion.

## Ethics Statement

The institutional review board of the Clinical Research Ethics Committee (CEIm) from Vall d'Hebron University Hospital approved the study (PR(AG)259/2020). The need for informed consent was waived by CEIm Vall d'Hebron University Hospital, as the study used data routinely collected for surveillance activities. All methods were performed in accordance with relevant guidelines and regulations.

## Conflicts of Interest

The authors declare no conflicts of interest.

## Supporting information

Campos et al 2024 Supplementary Material.

## Data Availability

Sequences from the Omicron variants BA.1, BA.1.1, BA.2, BA.5, BQ.1.1, XBB.1.5, and BA.2.86 were uploaded to the GeneBank Sequence Read Archive (SRA) database with BioProject accession number PRJNA1134434. Sequences from B.1.5, B.1.1, B.1.177, Alpha, Beta, and Delta variants were previously uploaded with accession number PRJNA788442, and sequences from Omicron B.1.1.529 BA.1 (12/2021) with accession number PRJNA1134434. Additionally, the FASTA files with the haplotypes and frequencies, will be made available upon request by contacting either of the corresponding authors via e‐mail. Sequences from the Omicron variants BA.1, BA.1.1, BA.2, BA.5, BQ.1.1, XBB.1.5, and BA.2.86 were uploaded to the GeneBank Sequence Read Archive (SRA) database with BioProject accession number PRJNA1134434. Sequences from B.1.5, B.1.1, B.1.177, Alpha, Beta, and Delta variants were previously uploaded with accession number PRJNA788442, and sequences from Omicron B.1.1.529 BA.1 (12/2021) with accession number PRJNA1134434. Additionally, the FASTA files with the haplotypes and frequencies, will be made available upon request by contacting either of the corresponding authors via e‐mail.
