# Supplementary material for: Deep Sequencing Reveals Dual Evolution of SARS‐CoV‐2: Insights Into Defective Genomes From Wuhan‐Hu‐1 Variants to Omicron Subvariants
Source: J Med Virol. 2025 Jun 30;97(7):e70476. doi: 10.1002/jmv.70476 (PMC12208013; doi:10.1002/jmv.70476)
Supplement: Supplementary file 1 — Campos et al 2024 Supplementary Material. [file JMV-97-e70476-s001.docx]

**Supplementary Table 1.** Patients clinical data and the corresponding GISAID ID codes for consensus sequences. Patients clinical data and the corresponding GISAID ID codes for consensus sequences. All samples were naso/oropharyngeal exudates from assymptomatic patients attended in primary care. SARS-CoV-2 RNA was detected using COBAS 5800 Technique, without Ct being available.

| **PATIENT ID** | **GISAID ID** | **LINEAGE** | **AGE** | **EXTRACTION DATE** | **GENDER M o F** |
| --- | --- | --- | --- | --- | --- |
| **P01** | EPI_ISL_10128049 | **BA.1** | 84 yo | 07/02/2022 | M |
| **P02** | EPI_ISL_10128086 |  | 20 yo | 08/02/2022 | F |
| **P03** | EPI_ISL_10128100 |  | 22 yo | 09/02/2022 | M |
| **P04** | EPI_ISL_10128069 |  | 33 yo | 11/02/2022 | F |
| **P05** | EPI_ISL_10740056 |  | 19 yo | 25/02/2022 | M |
| **P06** | EPI_ISL_10128090 |  | 29 yo | 07/02/2022 | M |
| **P07** | EPI_ISL_10128039 |  | 95 yo | 08/02/2022 | F |
| **P08** | EPI_ISL_10128103 |  | 31 yo | 11/02/2022 | F |
| **P09** | EPI_ISL_8790362 |  | 76 yo | 23/02/2022 | M |
| **P10** | EPI_ISL_10128054 |  | 87 yo | 08/02/2022 | M |
| **P11** | EPI_ISL_10128099 |  | 81 yo | 10/02/2022 | F |
| **P12** | EPI_ISL_10128082 |  | 39 yo | 12/02/2022 | M |
| **P13** | EPI_ISL_10740038 |  | 43 yo | 22/02/2022 | M |
| **P14** | EPI_ISL_9905096 | **BA.1.1** | 22 yo | 02/02/2022 | F |
| **P15** | EPI_ISL_9905067 |  | 59 yo | 03/02/2022 | M |
| **P16** | EPI_ISL_9905025 |  | 70 yo | 04/02/2022 | M |
| **P17** | EPI_ISL_10448850 |  | 31 yo | 15/02/2022 | M |
| **P18** | EPI_ISL_10448874 |  | 90 yo | 15/02/2022 | F |
| **P19** | EPI_ISL_9905029 |  | 6 yo | 01/02/2022 | M |
| **P20** | EPI_ISL_9905015 |  | 31 yo | 03/02/2022 | M |
| **P21** | EPI_ISL_10448862 |  | 53 yo | 15/02/2022 | M |
| **P22** | EPI_ISL_10448867 |  | 2 yo | 15/02/2022 | M |
| **P23** | EPI_ISL_9905032 |  | 34 yo | 01/02/2022 | F |
| **P24** | EPI_ISL_9905080 |  | 72 yo | 02/02/2022 | M |
| **P25** | EPI_ISL_10448825 |  | 40 yo | 15/02/2022 | F |
| **P26** | EPI_ISL_10448821 |  | 47 yo | 15/02/2022 | M |
| **P27** | EPI_ISL_10740044 |  | 23 yo | 15/02/2022 | M |
| **P28** | EPI_ISL_9905157 | **BA.2** | 4 yo | 02/02/2022 | M |
| **P29** | EPI_ISL_9905168 |  | 85 yo | 15/02/2022 | F |
| **P30** | EPI_ISL_10448860 |  | 98 yo | 15/02/2022 | F |
| **P31** | EPI_ISL_10448846 |  | 61 yo | 15/02/2022 | F |
| **P32** | EPI_ISL_10448844 |  | 26 yo | 14/02/2022 | F |
| **P33** | EPI_ISL_10448842 |  | 62 yo | 14/02/2022 | M |
| **P34** | NA |  | 23 yo | 15/02/2022 | F |
| **P35** | EPI_ISL_10448859 |  | 88 yo | 15/02/2022 | F |
| **P36** | EPI_ISL_10448822 |  | 26 yo | 15/02/2022 | M |
| **P37** | EPI_ISL_9904971 |  | 24 yo | 01/02/2022 | M |
| **P38** | EPI_ISL_10448843 |  | 23 yo | 14/02/2022 | F |
| **P39** | EPI_ISL_10448857 |  | 90 yo | 15/02/2022 | F |
| **P40** | EPI_ISL_10448852 |  | 29 yo | 15/02/2022 | F |
| **P41** | EPI_ISL_10448853 |  | 38 yo | 15/02/2022 | F |
| **P42** | EPI_ISL_13011215 | **BA.5** | 27 yo | 24/05/2022 | F |
| **P43** | EPI_ISL_13011218 |  | 46 yo | 24/05/2022 | F |
| **P44** | EPI_ISL_13134282 |  | 22 yo | 30/05/2022 | M |
| **P45** | EPI_ISL_13134277 |  | 30 yo | 30/05/2022 | F |
| **P47** | EPI_ISL_13244325 |  | 58 yo | 30/05/2022 | F |
| **P48** | EPI_ISL_13244346 |  | 41 yo | 07/06/2022 | M |
| **P49** | EPI_ISL_13244343 |  | 71 yo | 07/06/2022 | F |
| **P50** | EPI_ISL_13358839 |  | 51 yo | 08/06/2022 | M |
| **P51** | EPI_ISL_13358878 |  | 55 yo | 13/06/2022 | M |
| **P52** | EPI_ISL_13358877 |  | 64 yo | 13/06/2022 | M |
| **P53** | EPI_ISL_13477330 |  | 15 yo | 14/06/2022 | F |
| **P54** | EPI_ISL_13477343 |  | 60 yo | 15/06/2022 | M |
| **P55** | EPI_ISL_13477306 |  | 87 yo | 20/06/2022 | M |
| **P56** | EPI_ISL_15927217 | **BQ.1.1** | 95 yo | 16/11/2022 | F |
| **P57** | EPI_ISL_15927216 |  | 83 yo | 16/11/2022 | M |
| **P58** | EPI_ISL_15927223 |  | 98 yo | 18/11/2022 | F |
| **P59** | EPI_ISL_15927209 |  | 49 yo | 18/11/2022 | F |
| **P60** | EPI_ISL_15927199 |  | 90 yo | 18/11/2022 | F |
| **P61** | EPI_ISL_15927198 |  | 91 yo | 18/11/2022 | F |
| **P62** | EPI_ISL_15927161 |  | 30 yo | 22/11/2022 | M |
| **P63** | EPI_ISL_15927183 |  | 42 yo | 22/11/2022 | F |
| **P64** | EPI_ISL_15927175 |  | 43 yo | 22/11/2022 | M |
| **P65** | EPI_ISL_15927178 |  | 52 yo | 22/11/2022 | F |
| **P66** | EPI_ISL_15927179 |  | 67 yo | 22/11/2022 | M |
| **P67** | EPI_ISL_15927181 |  | 64 yo | 22/11/2022 | M |
| **P68** | EPI_ISL_15927184 |  | 55 yo | 22/11/2022 | F |
| **P69** | EPI_ISL_15927170 |  | 86 yo | 23/11/2022 | F |
| **P70** | EPI_ISL_15927171 |  | 86 yo | 23/11/2022 | F |

**Supplementary Table 2.** Number of reads obtained from amplicon A72 to amplicon A84 (v4.1, ARTIC Network), per patient. Coloured numbers indicate awful (under 1K reads), poor (from 1K to 10K reads), acceptable (from 10K to 30K reads), notable (from 30K to 80K reads) or excellent (over 80k) depths. Total number of reads per variant are shown.

| **Variant** | **Patient** | **A72** | **A73** | **A74** | **A75** | **A76** | **A77** | **A78** | **A79** | **A80** | **A81** | **A82** | **A83** | **A84** |  |  |  |  |
| --- | --- | --- | --- | --- | --- | --- | --- | --- | --- | --- | --- | --- | --- | --- | --- | --- | --- | --- |
| **Omicron BA.1** | **P01** | 61460 | 92633 | 91729 | 64176 | 92453 | 85798 | 97418 | 136096 | 118483 | 120926 | 125551 | 104847 | 92034 |  |  | OVER 80K |  |
|  | **P02** | 61772 | 76540 | 63914 | 61064 | 91463 | 85748 | 95138 | 93813 | 87712 | 98659 | 102816 | 93585 | 68596 |  |  | 30K-80K |  |
|  | **P03** | 69658 | 74522 | 103199 | 55229 | 114807 | 79429 | 109906 | 85999 | 108803 | 95623 | 118755 | 94858 | 113631 |  |  | 10K - 30K |  |
|  | **P04** | 51678 | 68857 | 63946 | 52461 | 84403 | 77855 | 85426 | 78843 | 75997 | 84457 | 87902 | 83058 | 64645 |  |  | 1K - 10K |  |
|  | **P05** | 48967 | 106907 | 68546 | 72368 | 79294 | 102783 | 76767 | 130007 | 71253 | 136375 | 78657 | 124695 | 83732 |  |  | UNDER 1K |  |
|  | **P06** | 31247 | 81975 | 39559 | 54319 | 46034 | 81424 | 50817 | 89325 | 47316 | 110227 | 55209 | 92520 | 49823 |  |  |  |  |
|  | **P07** | 45170 | 76498 | 46159 | 26345 | 66911 | 76442 | 69457 | 87816 | 58256 | 103423 | 65038 | 79204 | 70487 |  |  |  |  |
|  | **P08** | 47631 | 59369 | 52771 | 30639 | 69342 | 61589 | 73735 | 61425 | 67136 | 76891 | 79778 | 65326 | 69401 |  |  | **Subvariant** | **Reads** |
|  | **P09** | 49528 | 75843 | 53336 | 39680 | 65327 | 81706 | 65104 | 73975 | 60186 | 97219 | 78090 | 75174 | 52418 |  |  | **BA.1** | 12854977 |
|  | **P10** | 50943 | 76630 | 55830 | 48336 | 63090 | 71991 | 74491 | 88607 | 65805 | 102537 | 77813 | 91531 | 79033 |  |  | **BA.1.1** | 14103185 |
|  | **P11** | 39702 | 89321 | 38749 | 58503 | 63300 | 120862 | 68993 | 116706 | 41863 | 120476 | 44951 | 113646 | 66721 |  |  | **BA.2** | 14909443 |
|  | **P12** | 59633 | 57290 | 55980 | 39653 | 82403 | 71640 | 84368 | 64290 | 68971 | 79113 | 71430 | 76175 | 78932 |  |  | **BA.5** | 14727193 |
|  | **P13** | 51390 | 78264 | 45896 | 50585 | 63376 | 78431 | 67525 | 94614 | 58520 | 105244 | 64651 | 91801 | 72030 |  |  | **BQ.1.1** | 10987581 |
|  | **Total reads** | **668779** | **1014649** | **779614** | **653358** | **982203** | **1075698** | **1019145** | **1201516** | **930301** | **1331170** | **1050641** | **1186420** | **961483** | **12854977** |  | **XBB.1.5** | 20608833 |
| **Omicron BA.1.1** | **P14** | 66200 | 82978 | 69782 | 53198 | 99636 | 99710 | 105500 | 100129 | 88579 | 104040 | 98081 | 101589 | 79942 |  |  | **BA.2.86** | 18166960 |
|  | **P15** | 51449 | 75162 | 66864 | 48720 | 94077 | 91823 | 101486 | 98487 | 85416 | 97472 | 94280 | 92822 | 82540 |  |  | **Total reads** | **106.358.172** |
|  | **P16** | 50501 | 93346 | 84424 | 60509 | 82487 | 79000 | 94258 | 125789 | 102249 | 123276 | 121625 | 105876 | 85761 |  |  |  |  |
|  | **P17** | 74257 | 38434 | 83556 | 31089 | 118635 | 60768 | 130540 | 52088 | 108850 | 61567 | 125033 | 64222 | 90596 |  |  |  |  |
|  | **P18** | 48416 | 89377 | 70211 | 58443 | 82092 | 87866 | 91331 | 119898 | 86645 | 115869 | 99696 | 106165 | 80236 |  |  |  |  |
|  | **P19** | 41123 | 65675 | 51068 | 46910 | 55873 | 60095 | 64572 | 77832 | 56989 | 90195 | 73222 | 76365 | 68884 |  |  |  |  |
|  | **P20** | 32367 | 76558 | 36839 | 42523 | 48744 | 74752 | 50637 | 85374 | 44829 | 99748 | 57297 | 80884 | 50818 |  |  |  |  |
|  | **P21** | 73053 | 142372 | 77973 | 79551 | 109152 | 152216 | 113516 | 167856 | 102209 | 197814 | 125906 | 154941 | 85792 |  |  |  |  |
|  | **P22** | 30305 | 73721 | 35378 | 41847 | 41932 | 64981 | 47150 | 65430 | 41917 | 84759 | 49313 | 71383 | 25331 |  |  |  |  |
|  | **P23** | 42983 | 72545 | 58208 | 48383 | 66507 | 67777 | 78617 | 93909 | 69356 | 104606 | 81821 | 88005 | 82809 |  |  |  |  |
|  | **P24** | 50917 | 72912 | 46495 | 40938 | 65366 | 83894 | 68651 | 80423 | 54502 | 94602 | 59194 | 84118 | 70933 |  |  |  |  |
|  | **P25** | 29594 | 110256 | 36117 | 72692 | 40769 | 108673 | 46705 | 121837 | 39114 | 145948 | 44737 | 131426 | 52745 |  |  |  |  |
|  | **P26** | 36591 | 63557 | 51952 | 47127 | 55862 | 57204 | 64798 | 86262 | 59037 | 92033 | 69437 | 78224 | 71040 |  |  |  |  |
|  | **P27** | 55790 | 76753 | 58989 | 46093 | 74234 | 78421 | 77308 | 87750 | 70503 | 102577 | 78546 | 93234 | 79865 |  |  |  |  |
|  | **Total reads** | **683546** | **1133646** | **827856** | **718023** | **1035366** | **1167180** | **1135069** | **1363064** | **1010195** | **1514506** | **1178188** | **1329254** | **1007292** | **14103185** |  |  |  |
| **Omicron BA.2** | **P28** | 51803 | 154140 | 38309 | 27005 | 68795 | 166788 | 59413 | 144687 | 46368 | 214638 | 77971 | 149074 | 50359 |  |  |  |  |
|  | **P29** | 78867 | 66690 | 91886 | 18136 | 108989 | 79318 | 109189 | 78878 | 103364 | 88512 | 126171 | 89259 | 83319 |  |  |  |  |
|  | **P30** | 68433 | 87109 | 83763 | 25775 | 100120 | 90012 | 100992 | 99244 | 98308 | 110372 | 118469 | 107713 | 82461 |  |  |  |  |
|  | **P31** | 65951 | 52503 | 83142 | 16010 | 97976 | 90355 | 89527 | 88561 | 79103 | 96168 | 87639 | 97711 | 89353 |  |  |  |  |
|  | **P32** | 50852 | 81975 | 52984 | 46123 | 61611 | 81135 | 70810 | 101476 | 63316 | 109788 | 79646 | 90049 | 65617 |  |  |  |  |
|  | **P33** | 29945 | 52681 | 36823 | 21125 | 42325 | 47825 | 49495 | 63516 | 44211 | 68852 | 52190 | 57614 | P345 |  |  |  |  |
|  | **P34** | 113987 | 212200 | 150083 | 99259 | 160386 | 185819 | 202701 | 283361 | 179819 | 290258 | 213880 | 234120 | 206195 |  |  |  |  |
|  | **P35** | 43981 | 73317 | 52157 | 32101 | 52351 | 62586 | 69282 | 118416 | 59268 | 111670 | 73195 | 79827 | 70511 |  |  |  |  |
|  | **P36** | 45514 | 40142 | 68069 | 7867 | 70481 | 48803 | 101542 | 40355 | 74985 | 47120 | 89179 | 45037 | 93615 |  |  |  |  |
|  | **P37** | 40636 | 60882 | 52186 | 27379 | 56024 | 55741 | 67880 | 75499 | 55843 | 81551 | 65093 | 71707 | 70531 |  |  |  |  |
|  | **P38** | 56828 | 106753 | 62772 | 61256 | 73651 | 102348 | 69636 | 124334 | 63801 | 141517 | 73436 | 129664 | 78832 |  |  |  |  |
|  | **P39** | 43410 | 62363 | 43712 | 21590 | 59158 | 60334 | 58977 | 74620 | 49449 | 81818 | 54690 | 71082 | 62777 |  |  |  |  |
|  | **P40** | 48616 | 65144 | 46671 | 20580 | 83364 | 98764 | 88779 | 91263 | 52251 | 84980 | 55674 | 84918 | 85119 |  |  |  |  |
|  | **P41** | 39679 | 104901 | 36534 | 37618 | 61089 | 151915 | 64600 | 133942 | 36169 | 134758 | 36811 | 129559 | 61864 |  |  |  |  |
|  | **Total reads** | **778502** | **1220800** | **899091** | **461824** | **1096320** | **1321743** | **1202823** | **1518152** | **1006255** | **1662002** | **1204044** | **1437334** | **1100553** | **14909443** |  |  |  |
| **Omicron BA.5** | **P43** | 51284 | 113402 | 57872 | 61054 | 68141 | 96289 | 60280 | 127772 | 55429 | 133260 | 62819 | 117613 | 67624 |  |  |  |  |
|  | **P44** | 55881 | 83579 | 63086 | 41493 | 72111 | 72439 | 70721 | 94294 | 63536 | 100492 | 78715 | 89587 | 79750 |  |  |  |  |
|  | **P45** | 86504 | 148161 | 36564 | 118935 | 43489 | 90327 | 60120 | 78835 | 35748 | 109115 | 74993 | 41207 | 7372 |  |  |  |  |
|  | **P47** | 59623 | 99155 | 65737 | 41486 | 70891 | 80324 | 70990 | 102066 | 63148 | 114683 | 83866 | 92693 | 71269 |  |  |  |  |
|  | **P48** | 62869 | 108166 | 65419 | 43238 | 68694 | 93311 | 68169 | 106026 | 53719 | 121180 | 78157 | 96493 | 61840 |  |  |  |  |
|  | **P49** | 56603 | 111325 | 58974 | 58881 | 65406 | 98809 | 61762 | 114459 | 56086 | 125893 | 76812 | 109709 | 67102 |  |  |  |  |
|  | **P50** | 67207 | 125094 | 72654 | 46916 | 84062 | 111520 | 80714 | 140599 | 71853 | 151721 | 89479 | 128601 | 94045 |  |  |  |  |
|  | **P51** | 70670 | 117794 | 78913 | 48685 | 90949 | 97284 | 87826 | 138333 | 83212 | 140972 | 94303 | 120152 | 101648 |  |  |  |  |
|  | **P52** | 57722 | 84536 | 67155 | 31229 | 79201 | 75217 | 80773 | 95263 | 73991 | 101601 | 86409 | 89367 | 95258 |  |  |  |  |
|  | **P53** | 102268 | 195665 | 103553 | 103246 | 104537 | 169462 | 108223 | 190388 | 71153 | 227069 | 144831 | 152098 | 65947 |  |  |  |  |
|  | **P54** | 69654 | 121357 | 75079 | 43139 | 92145 | 105071 | 92515 | 155503 | 81604 | P4851 | 92845 | 129073 | 110798 |  |  |  |  |
|  | **P55** | 61488 | 151127 | 68077 | 55360 | 83300 | 136040 | 79066 | 179027 | 68876 | 191559 | 77513 | 162137 | 92389 |  |  |  |  |
|  | **P42** | 29025 | 138557 | 21573 | 90675 | 31165 | 114319 | 23189 | P4426 | 21686 | 147810 | 34223 | 97925 | 36010 |  |  |  |  |
|  | **Total reads** | **830798** | **1597918** | **834656** | **784337** | **954091** | **1340412** | **944348** | **1522565** | **800041** | **1665355** | **1074965** | **1426655** | **951052** | **14727193** |  |  |  |
| **Omicron BQ.1.1** | **P61** | 36504 | 59286 | 52210 | 28087 | 68155 | 65132 | 61033 | 72668 | 52141 | 80276 | 64745 | 76646 | 64589 |  |  |  |  |
|  | **P60** | 32270 | 51006 | 46199 | 22795 | 68949 | 70693 | 58510 | 56840 | 44565 | 66622 | 57412 | 65874 | 60519 |  |  |  |  |
|  | **P59** | 33902 | 60673 | 52545 | 28386 | 69592 | 68149 | 61719 | 67062 | 52451 | 80728 | 63032 | 74613 | 68061 |  |  |  |  |
|  | **P63** | 35872 | 63275 | 48896 | 35142 | 62393 | 70879 | 53769 | 65606 | 37769 | 83834 | 66641 | 78840 | 54908 |  |  |  |  |
|  | **P70** | 27888 | 75389 | 39302 | 41137 | 47543 | 85805 | 39902 | 84942 | 36304 | 99024 | 44032 | 96458 | 46378 |  |  |  |  |
|  | **P69** | 36622 | 58430 | 45096 | 28800 | 61354 | 67504 | 50872 | 64048 | 40315 | 74520 | 55395 | 73302 | 55327 |  |  |  |  |
|  | **P58** | 23440 | 51010 | 47673 | 23144 | 56746 | 54881 | 53979 | 62057 | 48423 | 75040 | 55576 | 68844 | 56842 |  |  |  |  |
|  | **P62** | 30243 | 57795 | 47072 | 30541 | 60943 | 63655 | 53140 | 61545 | 44380 | 77406 | 65437 | 73033 | 58010 |  |  |  |  |
|  | **P57** | 31105 | 63080 | 47791 | 27031 | 65349 | 84484 | 59798 | 58732 | 36121 | 77279 | 59277 | 72037 | 54520 |  |  |  |  |
|  | **P56** | 41536 | 66645 | 51711 | 30374 | 65017 | 66195 | 57235 | 70442 | 45998 | 83997 | 65929 | 79351 | 63220 |  |  |  |  |
|  | **P67** | 36795 | 60470 | 45579 | 24467 | 65923 | 68046 | 59995 | 72720 | 46574 | 75766 | 52935 | 76721 | 62786 |  |  |  |  |
|  | **P66** | 35023 | 62585 | 50655 | 27669 | 64449 | 72311 | 56254 | 61690 | 45524 | 73894 | 57470 | 74094 | 58082 |  |  |  |  |
|  | **P65** | 32428 | 59756 | 50656 | 24429 | 66253 | 65800 | 62830 | 63065 | 50714 | 72251 | 62965 | 71455 | 62624 |  |  |  |  |
|  | **P64** | 33001 | 67129 | 52582 | 24369 | 64856 | 79911 | 64075 | 64466 | 41229 | 85431 | 69415 | 71880 | 59632 |  |  |  |  |
|  | **P68** | 27502 | 46579 | 35950 | 10358 | 49357 | 54497 | 44605 | 50392 | 34241 | 59460 | 41389 | 51873 | 46438 |  |  |  |  |
|  | **Total reads** | **494131** | **903108** | **713917** | **406729** | **936879** | **1037942** | **837716** | **976275** | **656749** | **1165528** | **881650** | **1105021** | **871936** | **10987581** |  |  |  |
| **Omicron XBB.1.5** | **P71** | 67650 | 119006 | 86395 | 62698 | 115577 | 127249 | 97667 | 105798 | 92235 | 194963 | 103227 | 157244 | 97841 |  |  |  |  |
|  | **P72** | 68193 | 115080 | 85881 | 58130 | 109678 | 121773 | 101749 | 116532 | 94241 | 200956 | 103140 | 165389 | 108793 |  |  |  |  |
|  | **P73** | 69876 | 108535 | 88340 | 55287 | 109413 | 109106 | 97055 | 101431 | 92575 | 166560 | 104853 | 140847 | 101971 |  |  |  |  |
|  | **P74** | 61501 | 104590 | 78909 | 52658 | 98583 | 101290 | 93463 | 126377 | 92675 | 181490 | 104669 | 151337 | 109237 |  |  |  |  |
|  | **P75** | 68288 | 98116 | 79317 | 40728 | 104301 | 110704 | 91029 | 95619 | 88163 | 142716 | 95995 | 128463 | 97028 |  |  |  |  |
|  | **P76** | 56267 | 118077 | 64513 | 55074 | 88516 | 130023 | 79735 | 114916 | 75694 | 163107 | 82503 | 154394 | 86140 |  |  |  |  |
|  | **P77** | 69834 | 131796 | 83537 | 55688 | 108920 | 121137 | 99774 | 133361 | 89890 | 174691 | 98642 | 152657 | 109519 |  |  |  |  |
|  | **P78** | 59891 | 122862 | 78584 | 53341 | 100327 | 114979 | 91396 | 125981 | 86543 | 170868 | 92048 | 152986 | 100732 |  |  |  |  |
|  | **P79** | 63207 | 126936 | 77872 | 46590 | 100242 | 115897 | 96808 | 139315 | 88110 | 169245 | 99017 | 150305 | 104797 |  |  |  |  |
|  | **P80** | 71824 | 109383 | 89522 | 34950 | 113825 | 95163 | 98217 | 115902 | 89829 | 142240 | 99509 | 121789 | 110033 |  |  |  |  |
|  | **P81** | 65779 | 128745 | 81738 | 37014 | 103082 | 120967 | 90547 | 142285 | 85212 | 164776 | 92182 | 141450 | 106129 |  |  |  |  |
|  | **P82** | 36902 | 111609 | 71874 | 46751 | 90594 | 106746 | 90437 | 127684 | 80628 | 148738 | 89445 | 134263 | 98257 |  |  |  |  |
|  | **P83** | 69522 | 171848 | 80163 | 86153 | 106408 | 159142 | 98848 | 184067 | 96339 | 241424 | 105569 | 201835 | 99651 |  |  |  |  |
|  | **P84** | 76154 | 112591 | 98000 | 53788 | 124268 | 112456 | 113784 | 112142 | 98746 | 153024 | 110408 | 136426 | 122299 |  |  |  |  |
|  | **P85** | 69056 | 123051 | 80892 | 65135 | 106800 | 118081 | 88001 | 111210 | 80500 | 170819 | 94742 | 138275 | 103732 |  |  |  |  |
|  | **Total reads** | **973944** | **1802225** | **1225537** | **803985** | **1580534** | **1764713** | **1428510** | **1852620** | **1331380** | **2585617** | **1475949** | **2227660** | **1556159** | **20608833** |  |  |  |
| **Omicron BA.2.86** | **P86** | 61060 | 234455 | 79073 | 123794 | 102557 | 253842 | 98067 | 301873 | 81645 | 337687 | 96001 | 325567 | 105304 |  |  |  |  |
|  | **P87** | 83544 | 216894 | 100249 | 130958 | 122493 | 281081 | 119044 | 289275 | 95920 | 328641 | 116263 | 320603 | 126320 |  |  |  |  |
|  | **P88** | 83071 | 133362 | 99539 | 40780 | 119467 | 285822 | 124111 | 289929 | 86883 | 378926 | 117611 | 269112 | 121543 |  |  |  |  |
|  | **P89** | 73047 | 301247 | 97385 | 142485 | 119470 | 322543 | 117034 | 374431 | 97905 | 415773 | 112297 | 402273 | 122884 |  |  |  |  |
|  | **P90** | 79478 | 224917 | 109911 | 119856 | 134280 | 238523 | 128925 | 274232 | 109135 | 299160 | 127698 | 305610 | 140854 |  |  |  |  |
|  | **P91** | 75140 | 222997 | 98260 | 104200 | 118700 | 228286 | 120716 | 263591 | 88435 | 287701 | 117956 | 279165 | 123116 |  |  |  |  |
|  | **P92** | 76844 | 195721 | 91238 | 99313 | 113758 | 226835 | 111022 | 237328 | 90884 | 264342 | 109350 | 262138 | 118725 |  |  |  |  |
|  | **P93** | 79567 | 239992 | 109486 | 128691 | 133551 | 274643 | 125946 | 275525 | 106086 | 327158 | 122118 | 310438 | 134279 |  |  |  |  |
|  | **Total reads** | **611751** | **1769585** | **785141** | **890077** | **964276** | **2111575** | **944865** | **2306184** | **756893** | **2639388** | **919294** | **2474906** | **993025** | **18166960** |  |  |  |

**Supplementary Table 3.** Defective haplotypes detected per variant and their corresponding residue position loss, number of lost nucleotides, total number of reads accounting for the deletion, population frequency and median of frequency of defective reads.

| **Variant** | **Deletion name** | **Amplicon** | **Δ (deleted nt positions)** | **aa** | **Δ size (nts deleted)** | **Patients** | **reads Δ** | **Total reads** | **Population frequency (%)** | **Median of frequency of defective reads** |
| --- | --- | --- | --- | --- | --- | --- | --- | --- | --- | --- |
| **Omicron (B.1.1.529) BA.1 (12/2021)** | **Δ110L** | A72 | 329-330 | Δ110L | 2 | **V100S01** | 20 | 4859 | **0.41** | **0.31** |
|  | **Δ246R-249L** | A73 | 736-745 | Δ246R-249L | 10 | **V100S06** | 1,299 | 18474 | **7.03** |  |
|  | **Δ346R** | A75 | 1036-1037 | Δ346R | 2 | **V100S04** | 23 | 10104 | **0.23** |  |
|  | **Δ396Y-397A** | A75 | 1187-1190 | Δ396Y-397A | 4 | **V100S08** | 36 | 8578 | **0.42** |  |
|  | **Δ397A-398D** | A75 | 1191-1192 | Δ397A-398D | 2 | **V98S12** | 20 | 14510 | **0.14** |  |
|  | **Δ474Q-475A** | A76 | 1421-1422 | Δ474Q-475A | Δ2 | **V100S08** | 190 | 32540 | **0.58** |  |
|  | **Δ594G** | A77 | 1779-1780 | Δ594G | 2 | **V100S08** | 157 | 119784 | **0.13** |  |
|  | **Δ640S-674S** | A78 | 1920-2021 | Δ640S-674S | 2 to 100 | **V98S06,** **V100S08,** **V98S12,** **V100S01** | 932 | 152298 | **0.69** |  |
|  | **Δ805I** | A79 | 2413-2414 | Δ805I | 2 | **V100S08** | 187 | 99604 | **0.19** |  |
|  | **Δ851F-873Y** | A80 | 2564-2632 | Δ851F-873Y | 53 to 69 | **V98S12** | 22 | 1239 | **1.76** |  |
|  | **Δ867A-894F** | A80 | 2612-2693 | Δ867A-894F | 82 | **V98S06** | 13 | 7126 | **0.18** |  |
|  | **Δ890L-912L** | A80 | 2681-2748 | Δ890L-912L | 68 | **V98S06** | 25 | 7126 | **0.35** |  |
|  | **Δ1007Y/H - 1008V** | A81 | 3018-3019 | Δ1007Y/H - 1008V | 2 | **V100S08** | 144 | 84466 | **0.17** |  |
|  | **Δ1028K** | A81 | 3080-3081 | Δ1028K | 2 | **V100S01** | 739 | 111880 | **0.66** |  |
|  | **Δ1062F** | A82 | 3185-3186 | Δ1062F | 2 | **V100S08** | 284 | 122901 | **0.23** |  |
|  | **Δ1075F-1076T** | A82 | 3225-3226 | Δ1075F-1076T | 2 | **V100S08** | 295 | 122901 | **0.24** |  |
|  | **Δ1087A-1099G** | A82 | 3260-3296 | Δ1087A-1099G | 60 | **V100S08** | 385 | 122901 | **0.31** |  |
| **Omicron BA.1 (02/2022)** | **Δ21R** | A72 | 61-62 | 21R | 2 | **P12** | 82 | 59633 | **0.14** | **0.22** |
|  | **Δ131C-132E** | A73 | 391-394 | 131C-132E | 5 | **P12, P02** | 187 | 133830 | **0.14** |  |
|  | **Δ169E-170Y** |  | 507-508 | 169E-170Y | 2 | **P02** | 127 | 76540 | **0.17** |  |
|  | **Δ194F-203I** |  | 582-608 | 194F-203I | 27 | **P12, P02** | 420 | 133830 | **0.31** |  |
|  | **Δ210I-212L** |  | 630-634 | 210I-212L | 5 | **P08** | 484 | 59369 | **0.82** |  |
|  | **Δ193V-194F** | A74 | 578-581 | 193V-194F | 4 | **P02** | 146 | 63914 | **0.22** |  |
|  | **Δ244L-245H** |  | 732-734 | 244L-245H | 4 | **P09** | 147 | 53336 | **0.28** |  |
|  | **Δ289V-290D** |  | 867-868 | 289V-290D | 2 | **P12** | 87 | 55980 | **0.16** |  |
|  | **Δ508Y** | A76 | 1532-1533 | 508Y | 2 | **P02** | 133 | 91463 | **0.15** |  |
|  | **Δ489Y-491P** | A77 | 1476-1480 | 489Y-491P | 5 | **P02** | 137 | 85748 | **0.16** |  |
|  | **Δ570A-571D** | A78 | 1719-1720 | 570A-571D | 2 | **P12** | 123 | 84368 | **0.15** |  |
|  | **Δ654E-674Y** |  | 1970-2029 | 654E-674Y | 60 | **P12, P08, P04** | 861 | 159161 | **0.54** |  |
|  | **Δ675Q** |  | 2032-2033 | 675Q | 2 | **P04** | 310 | 85426 | **0.36** |  |
|  | **Δ817F-821L** | A80 | 2460-2472 | 817F-821L | 13 | **P09** | 209 | 60186 | **0.35** |  |
|  | **Δ859T-860V** |  | 2584-2587 | 859T-860V | 4 | **P09** | 226 | 60186 | **0.38** |  |
| **Omicron BA.1.1** | **Δ55F** | A72 | 164-165 | 55F | 2 | **P21** | 211 | 73053 | **0.30** | **0.20** |
|  | **Δ110L** | A73 | 329-330 | 110L | 2 | **P20** | 140 | 76558 | **0,20** |  |
|  | **Δ154E-181G** |  | 462-543 | 154E-181G | 82 | **P17** | 367 | 180806 | **0.20** |  |
|  | **Δ196N-203I** |  | 587-608 | 196N-203I | 22 | **P17,** **P20** | 222 | 114992 | **0.20** |  |
|  | **Δ199G-200Y** | A74 | 597-600 | 199G-200Y | 4 | **P24** | 83 | 46495 | **0.18** |  |
|  | **Δ210I-212L** |  | 629-633 | 210I-212L | 5 | **P19** | 76 | 51068 | **0.15** |  |
|  | **Δ294D** |  | 879-880 | 294D | 2 | **P21** | 203 | 77973 | **0.26** |  |
|  | **Δ433V-434I** | A76 | 1308-1309 | 433V-434I | 2 | **P24** | 92 | 65366 | **0.14** |  |
|  | **Δ575A-576V** | A78 | 1734-1735 | 575A-576V | 2 | **P22** | 1369 | 47150 | **2.9** |  |
|  | **Δ626A-629L** |  | 1886-1896 | 626A-629L | 11 | **P24** | 84 | 68651 | **0.12** |  |
|  | **Δ817F-822L** | A80 | 2458-2475 | 817F-822L | 16 | **P27, P20** | 159 | 115332 | **0.14** |  |
|  | **Δ856N-860V** |  | 2575-2587 | 856N-860V | 13 | **P21** | 333 | 102209 | **0.33** |  |
|  | **Δ1133V** | A83 | 3407-3708 | 1133V | 2 | **P21** | 390 | 154941 | **0.25** |  |
| **Omicron BA.2** | **Δ110L** | A72 | 329-330 | 110L | 2 | **P39** | 75 | 43410 | **0.17** | **0.18** |
|  | **Δ231I-232G** | A74 | 693-694 | 231I-232G | 2 | **P31** | 212 | 83142 | **0.25** |  |
|  | **Δ242L-243A** |  | 725-729 | 242L-243A | 5 | **P39** | 160 | 43712 | **0.37** |  |
|  | **Δ621P-622V** | A78 | 1872-1873 | 621P-622V | 2 | **P31** | 152 | 89527 | **0.17** |  |
|  | **Δ744G** | A79 | 2233-2234 | 744G | 2 | **P29** | 106 | 78878 | **0.13** |  |
|  | **Δ818I-823F** | A80 | 2463-2476 | 818I-823F | 11 | **P39, P30** | 279 | 147757 | **0.19** |  |
|  | **Δ856N-860V** |  | 2576-2589 | 856N-860V | 14 | **P39, P30** | 269 | 147757 | **0.18** |  |
|  | **Δ1074N-1075F** | A86 | 3230-3234 | 1074N-1075F | 5 | **P29** | 132 | 89259 | **0.15** |  |
| **Omicron BA.5** | **Δ115Q** | A73 | 344-345 | 115Q | 2 | **P53** | 869 | 195665 | **0.44** | **0.19** |
|  | **Δ139P-144Y** |  | 357-362 | 139P-144Y | 14 | **P42** | 663 | 138557 | **0.48** |  |
|  | **Δ410I-411A** | A76 | 1239-1240 | 410I-411A | 2 | **P42** | 59 | 31165 | **0.19** |  |
|  | **Δ456F-468I** |  | 1375-1413 | 456F-468I | 37 | **P42, P44** | 328 | 168682 | **0.19** |  |
|  | **Δ467D** |  | 1409-1410 | 467D | 2 | **P49** | 98 | 65406 | **0.15** |  |
|  | **Δ856N-860V** | A80 | 2576-2589 | 856N-860V | 14 | **P48** | 84 | 53719 | **0.16** |  |
| **Omicron BQ.1.1** | **Δ110L** | A73 | 329-330 | Δ110L | 2 | **P62** | 109 | 57795 | **0.19** | **0.19** |
|  | **Δ541F-543F** | A77 | 1631-1638 | Δ543F-541F | 8 | **P62** | 113 | 63655 | **0.18** |  |
| **Omicron XBB.1.5** | **Δ110L** | A73 | 329-330 | 110L | 2 | **P85** | 385 | 123051 | **0.31** | **0.36** |
|  | **Δ118L-119I** |  | 354-355 | 118L-119I | 2 | **P85** | 424 | 123051 | **0.34** |  |
|  | **Δ145Y-146H** |  | 433-438 | 145Y-146H | 6 | **P71, P73, P75, P76, P78, P79, P82, P85** | 28512 | 928192 | **3.07** |  |
|  | **Δ286T-287D** | A74 | 858-859 | 286T-287D | 2 | **P85** | 358 | 80892 | **0.44** |  |
|  | **Δ361C-361C** | A75 | 1082-1083 | 361C-361C | 2 | **P85** | 234 | 65135 | **0.36** |  |
|  | **Δ368L-368L** |  | 1102-1103 | 368L-368L | 2 | **P85** | 197 | 65135 | **0.30** |  |
|  | **Δ393T-393T** |  | 1177-1178 | 393T-393T | 2 | **P85** | 177 | 65135 | **0.37** |  |
|  | **Δ1016A-1017E** | A82 | 3048-3049 | 1016A-1017E | 2 | **P85** | 340 | 94742 | **0.36** |  |
| **Omicron BA.2.86** | **Δ114T** | A72 | 341-342 | 114T | 2 | **P91, P92** | 207 | 158684 | **0.13** | **0.17** |
|  | **Δ110L** | A73 | 329-330 | 110L | 2 | **P87** | 366 | 216894 | **0.17** |  |
|  | **Δ199G-201F** | A74 | 596-602 | 199G-201F | 7 | **P88** | 189 | 99539 | **0.19** |  |
|  | **Δ244L** |  | 731-732 | 244L | 2 | **P88** | 488 | 99539 | **0.49** |  |
|  | **Δ429F-444K** | A76 | 1287-1330 | 429F-444K | 44 | **P88** | 231 | 119467 | **0.19** |  |
|  | **Δ618T-619E** | A78 | 1854-1855 | 618T-619E | 2 | **P88** | 158 | 124111 | **0.13** |  |
|  | **Δ939S-951V** | A81 | 2815-2851 | 939S-951V | 37 | **P87** | 419 | 328641 | **0.13** |  |
|  | **Δ1006T-1007Y** | A82 | 3018-3019 | 1006T-1007Y | 2 | **P88** | 212 | 117611 | **0.18** |  |
|  | **Δ1206Y-1240C** | A84 | 3618-3718 | 1206Y-1240C | 101 | **P87** | 159 | 126320 | **0.13** |  |

**Supplementary Figures 1A-1G.** Barr-plots of defective deletions found per variant. The x-axis displays the multiple alignment (MA) nucleotide positions and the amplitude of deletions across subregions, while the y-axis indicates the frequency of deletions (percentage) on the right and the number of reads on the left. Since no insertions causing defective genomes were observed, the MA positions correspond to the S gene positions. Dashed lines mark the S1/S2 (left) and S2’ (right) cleavage sites.


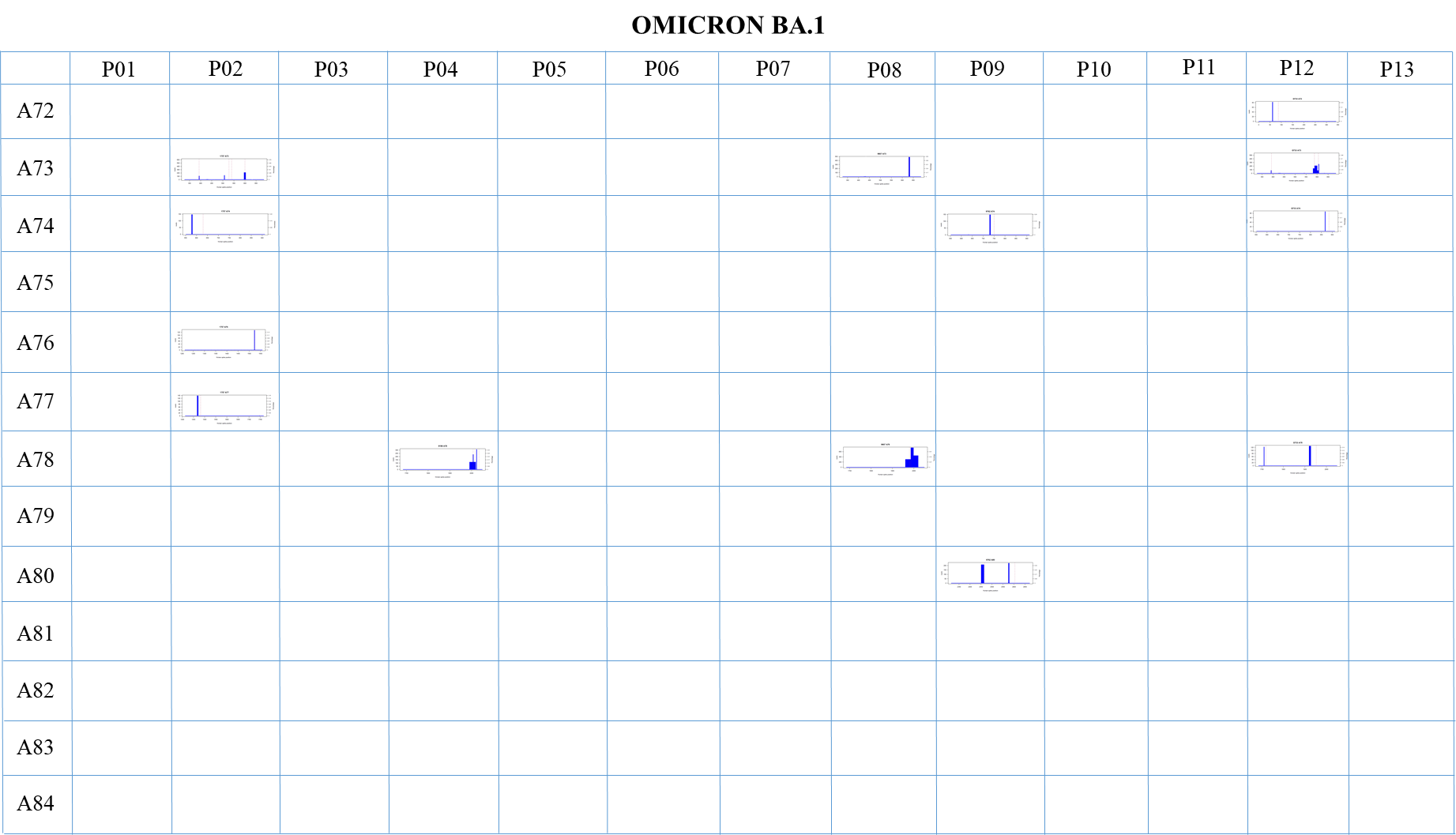
**Supplementary Figure 1A.** Omicron BA.1 bar-plots.

**
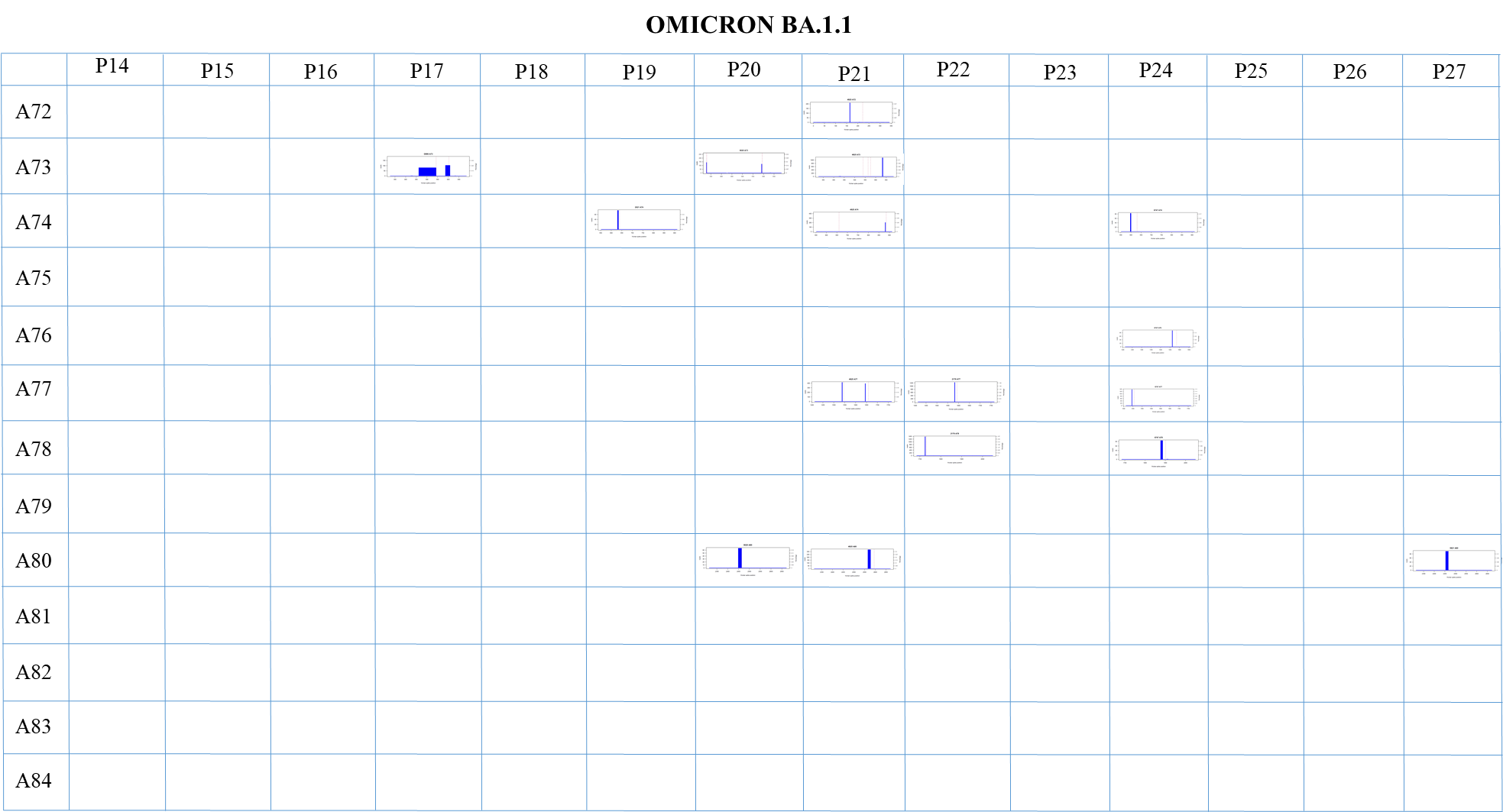
Supplementary Figure 1B.** Omicron BA.1.1 bar-plots.

**Supplementary Figure 1C.** Omicron BA.2 bar-plots.

**
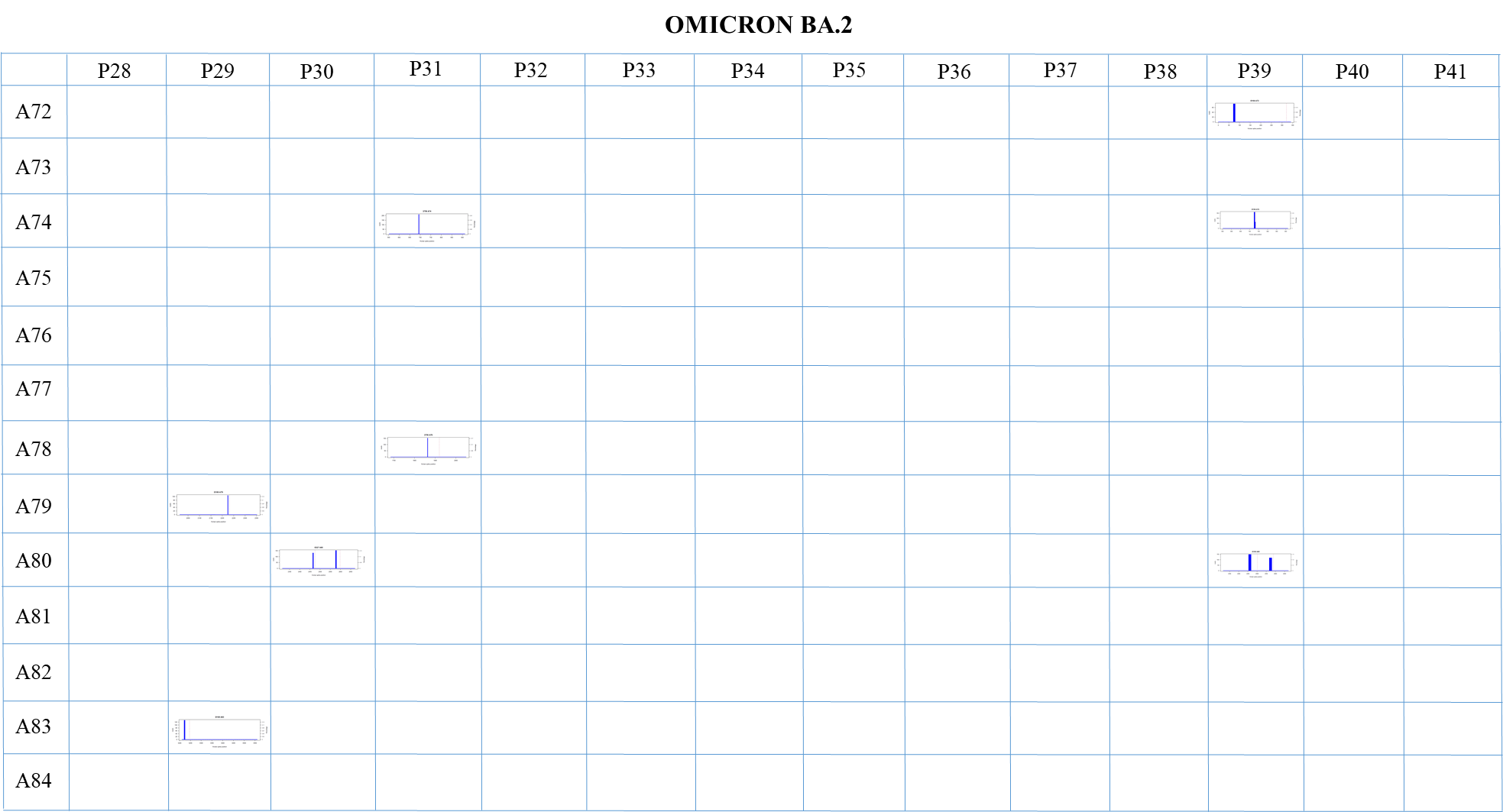
**
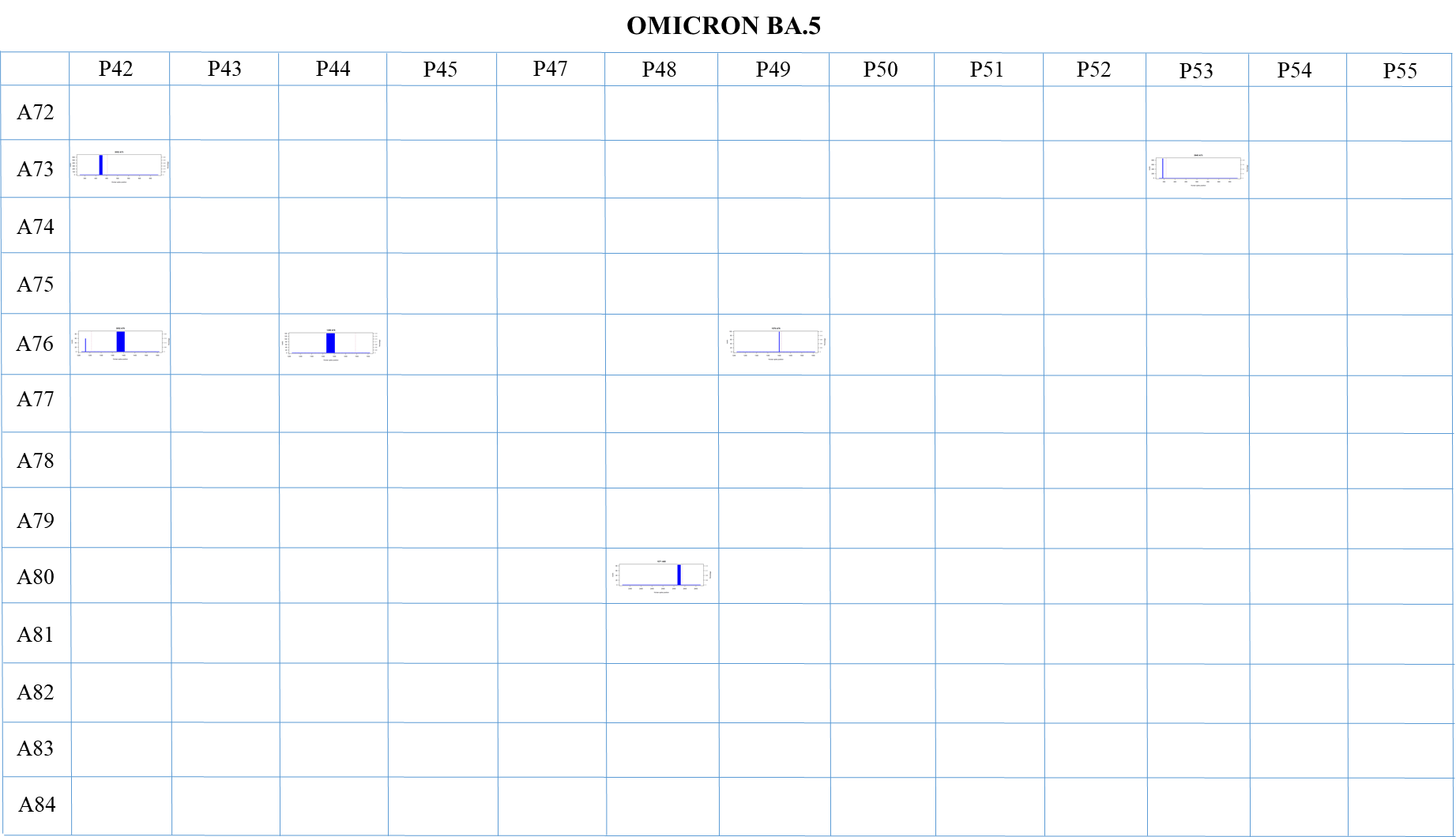
**Supplementary Figure 1D.** Omicron BA.5 bar-plots.

**Supplementary Figure 1E.** Omicron BQ.1.1 bar-plots.

**
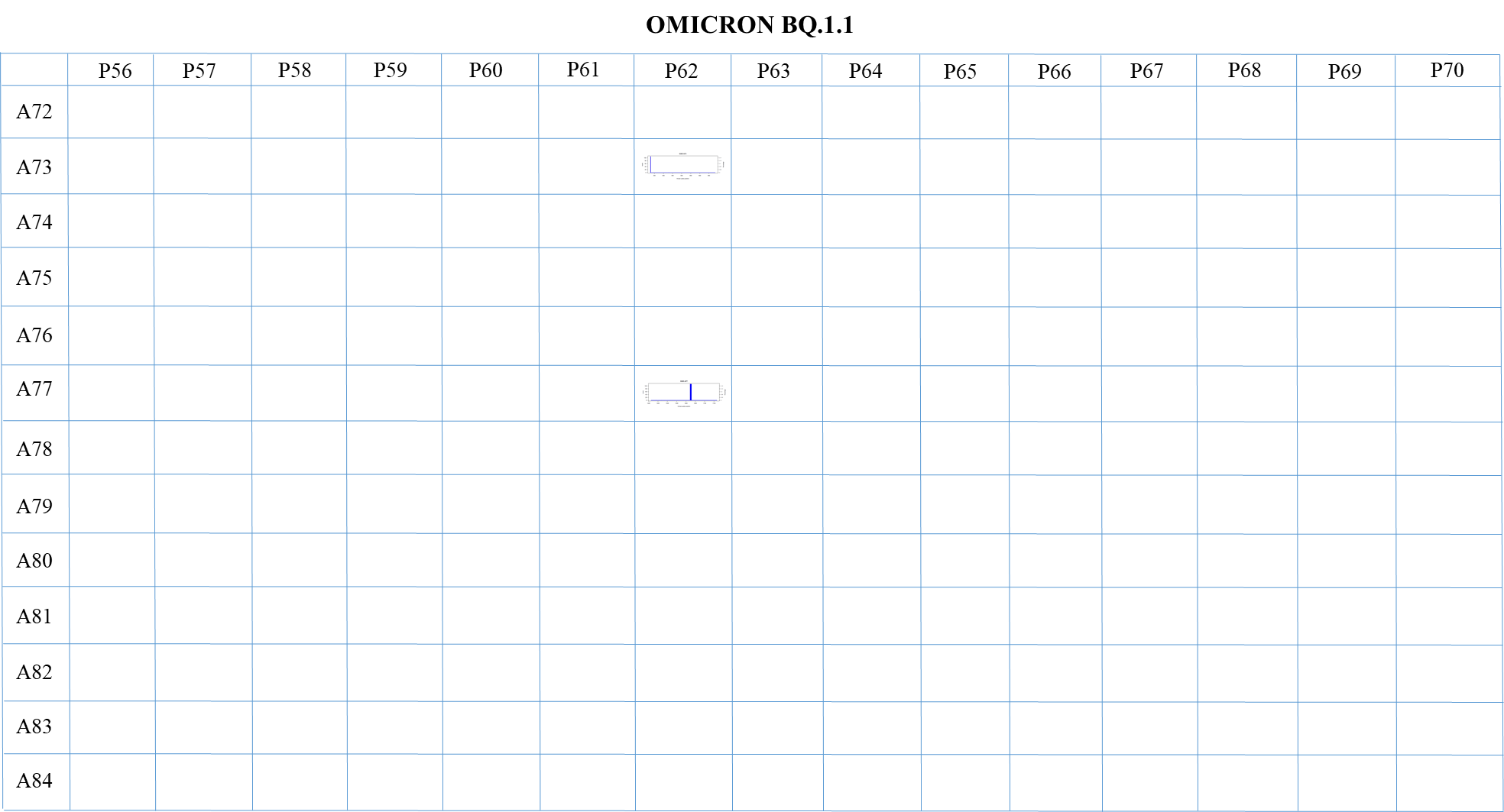

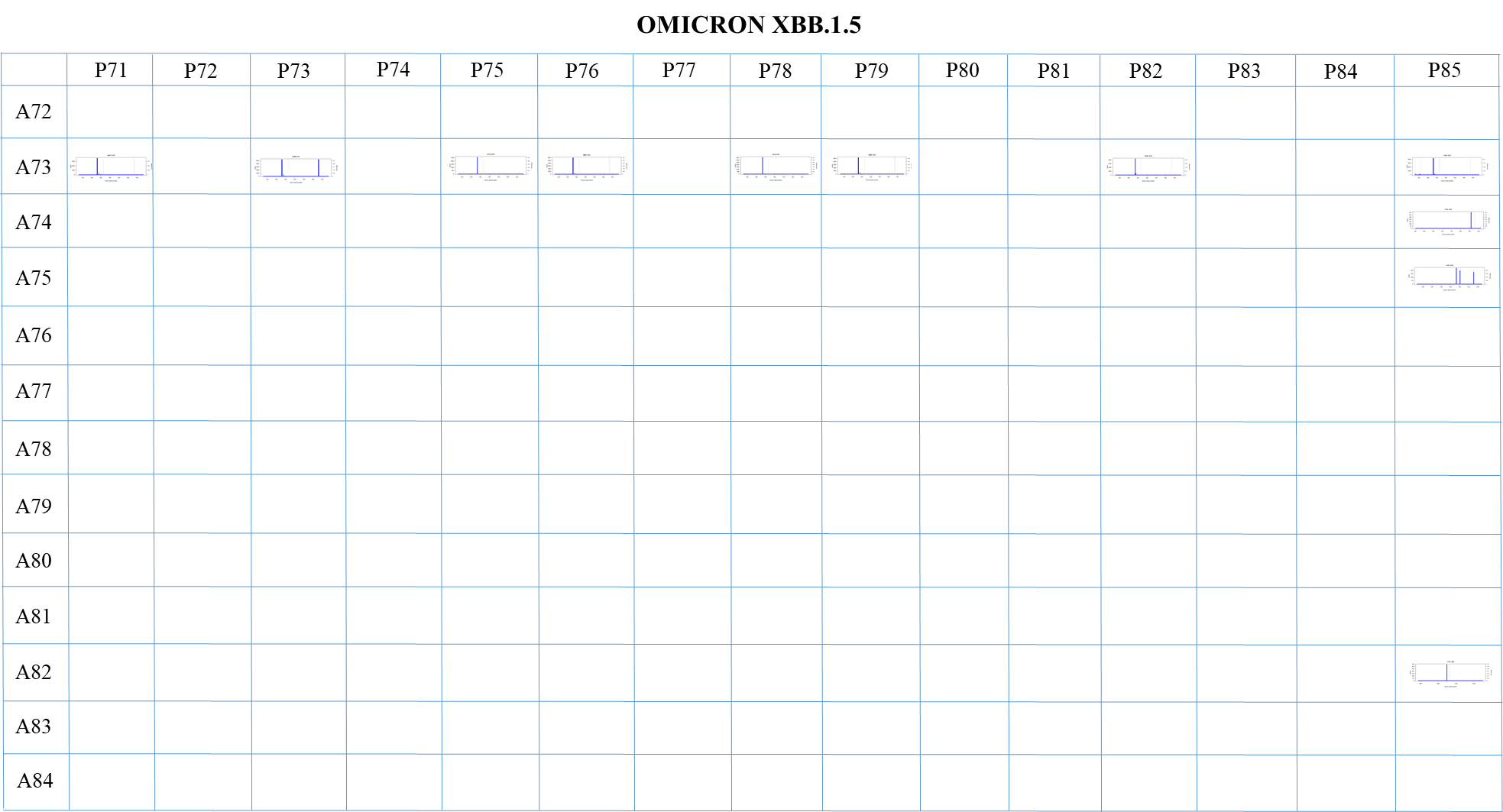
Supplementary Figure 1F.** Omicron XBB.1.5 bar-plots.


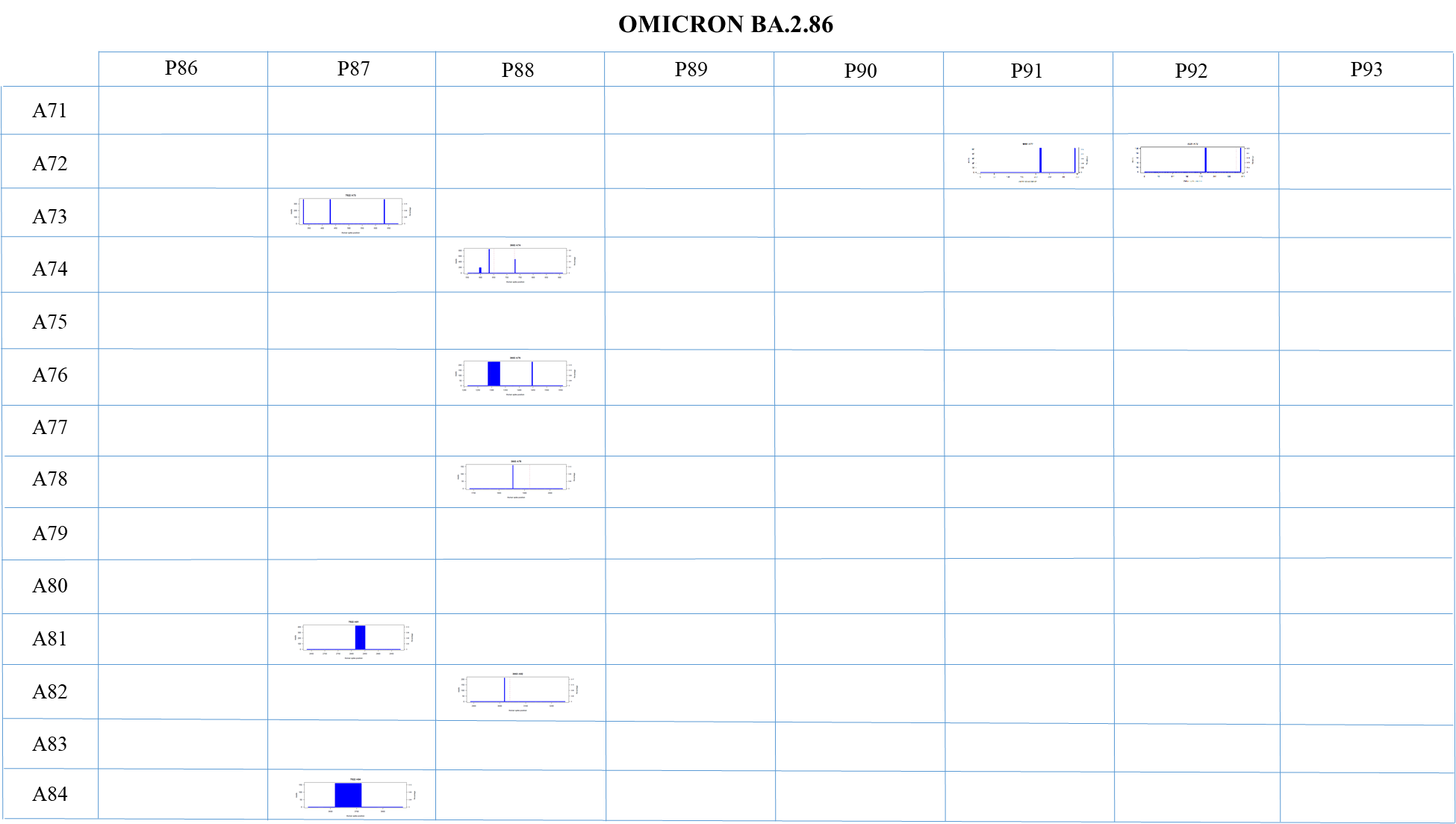
**Supplementary Figure 1G.** Omicron BA.2.86 bar-plots.
